# Exploring Consumers’ Situational Snacking Behaviors Using a Mobile App: Longitudinal Cohort Study (FOODLOOP) Among Millennials in the Netherlands

**DOI:** 10.2196/71858

**Published:** 2026-05-26

**Authors:** Mariëlle Tamara de Vaal, Vincenzo Fogliano, Ruud Verkerk, Bea L P A Steenbekkers

**Affiliations:** 1 Food Quality and Design (FQD), Wageningen University & Research, Bornse Weilanden 9, Wageningen, 6708 WG, The Netherlands, 31 317 480 100

**Keywords:** situational snacking, contextual eating, food choice motives, mobile app, ecological momentary assessment

## Abstract

**Background:**

Consumers are increasingly moving away from the traditional 3-meal-a-day eating routine to a pattern where they are snacking throughout the day to fulfill dietary needs, a trend known as “snackification.” Snacking depends on a variety of product-, context-, and consumer-specific determinants, but consumers’ long-term snacking behaviors in light of these determinants have remained little studied.

**Objective:**

This study aims to enhance understanding of consumers’ snacking behaviors by capturing longitudinal real-world situational snacking behaviors. As snackification is highly prominent in the Netherlands, and especially among millennials (born between 1980 and 2000), Dutch millennials were used as a case study.

**Methods:**

FOODLOOP studied situational snacking behaviors of a cohort of 264 Dutch millennials over the course of a year through a time-series structure. Data were collected on 12 nonconsecutive days, divided over the 4 seasons, using the mobile app Traqq (Department of Human Nutrition and Health of Wageningen University) and following the principles of ecological momentary assessment.

**Results:**

On average, 4.52 snacks were consumed per day, of which 64% were more healthful snacks, including coffee, tea, fruit, and bread products. Snacking was mostly driven by the food choice motives liking and appetite, as well as hunger and thirst, convenience, and pleasure. Most snacking occurred at home, with others, in the afternoon, and in spring. Dutch millennials with children consumed more snacks than Dutch millennials without children, and Dutch millennials born between 1980 and 1990 consumed more snacks than Dutch millennials born between 1990 and 2000.

**Conclusions:**

Our study shows that situational snacking determinants are essential for understanding consumers’ real-world snacking behaviors, as Dutch millennials’ snacking behaviors differ given product-, context-, and consumer-specific determinants, and are not restricted to specific motives, locations, social settings, or times, congruent with the ongoing snackification trend. We also demonstrate that a mobile app following the principles of ecological momentary assessment is a highly valuable methodological tool to gather longitudinal data on consumers’ situational snacking behaviors.

## Introduction

Consumers’ eating patterns have fundamentally changed in the past few years due to their increasingly busy lives. Many consumers are moving away from the traditional 3-meal-a-day pattern, consisting of breakfast, lunch, and dinner at predetermined times. Instead, consumers are moving toward a more flexible eating pattern, where snacking is becoming a new way of eating; consumers are increasingly having unplanned eating moments outside of meals, replacing meals with mini-meals and snacks, and snacking throughout the day to fulfill dietary needs [1-3]. This new eating pattern, where anything can be a snack, consumed anywhere, with anyone, and anytime, is known as “snackification” and is considered to stay [4,5].

An increase in snacking has often been linked to a poorer diet quality, body weight gain, and subsequent deterioration of health (cardiovascular diseases, obesity, etc), as snacks have traditionally been considered to consist of highly palatable “junk food” products (eg, cookies, confectionery, and chips), which contain high levels of calories, salt, sugar, and (saturated) fat [2,6]. In the new snackification frame, this view might be obsolete. Many consumers no longer merely snack on conventional highly palatable food products but on any type of food or beverage, including fruits, vegetables, and dairy products [3], which are tailored to different specific situations and support consumers’ health goals [7,8]. Therefore, snacking nowadays could also fit within a healthy dietary pattern and potentially benefit health by contributing to the daily intake of fibers, vitamins, minerals, and protein [3,7,9-13].

Snackification is a worldwide phenomenon, but the increase in snacking is especially pronounced in the Netherlands; the Dutch engage in, on average, 4-5 snacking occasions daily, which make up half of all daily consumption and 30% of the Dutch daily energy intake [14,15]. Snackification in the Netherlands, as well as in other countries, is mainly prominent among millennials [16,17], a generation born between 1980 and 2000 and characterized by the use and adaptation of technology [18]. Millennials have different life stages, undergoing significant lifestyle changes, such as leaving home, graduating, getting a job, and starting a family. Due to their increasingly busy lives and lifestyle changes, millennials often engage in snacking more than other generations [16] and seek snacks that support different situations and their health goals, in line with snackification [16,19,20]. In addition, millennials also influence the behaviors of other generations by spotting, adopting, and spreading consumer trends such as snackification [18]. Therefore, it is interesting to focus on millennials when studying snacking, since there is little knowledge to date about millennials’ snacking behaviors.

Multiple studies have been devoted to snacking. However, snacking and snacking-related concepts are often defined in different ways, using varying classifications (based on the time of day, type of food consumed, location of consumption, portion sizes, caloric values, nutrient quality, healthfulness, etc), therewith including or excluding certain products (eg, beverages, fruits, and vegetables) and consumption occasions (eg, meal replacements). This definition variability contributes to conflicting and incomparable findings around snacking behaviors and adds an additional uncertainty to the translation of scientific messages on snacking behaviors into effective snacking interventions, such as guidelines or recommendations [21-23]. To enable the formulation of successful snacking interventions, consumers’ snacking behaviors in different consumption situations need to be more fully understood [22,24].

Snacking, like any other dietary behavior, is situational; whether, what, where, when, with whom, how long, how, and how much to eat and/or drink can differ greatly in every snacking situation [14,25,26]. Snacking depends on a variety of determinants, including product-specific (eg, product type and portion size), context-specific (physical, social, and temporal contexts), and consumer-specific determinants. Consumer-specific determinants, including sociodemographic (eg, gender, age, socioeconomic status, education, and family composition) and lifestyle characteristics (health interest, activity level, diet, etc), also comprise mental processes, such as the motives for consumption [3,14,27-29]. These motives, known as food choice motives (FCM), are essential for understanding snacking behaviors and include physiological (eg, hunger or thirst), psychological (eg, affect regulation and pleasure), sociocultural (eg, traditional eating and sociability), practical (eg, convenience and choice limitation), and economic (eg, price) drivers, which shape everyday snacking consumption decisions [26,30].

Given the situational nature of snacking, there is a need for real-time, context-sensitive insights into consumers’ snacking behaviors. However, most snacking-related research focused on how snacking affects energy balance and weight status [3] or studied consumers’ snacking behaviors in isolation, in short time frames, and with a limited consideration of situational determinants [31]. To address this gap, we aim to provide a broader and more integrated understanding of consumers’ snacking behaviors by longitudinally exploring Dutch millennial consumers’ situational snacking behaviors given product-, context-, and consumer-specific determinants. We, therewith, extend prior work on snacking behaviors by capturing real-world snacking situations while accounting for variances given situational determinants.

Studying situational behaviors longitudinally poses a challenge in using suitable and effective methods for correct measurement and understanding [31]. Given recent advancements in mobile technologies and the proven benefits of apps for mobile health (mHealth) purposes (eg, facilitating dietary monitoring and supporting health education and policies), mobile apps are increasingly used among behavioral and nutrition researchers for answering key food and health-related research questions and therewith achieving greater understanding of dietary behaviors [26,31-34]. Mobile apps allow researchers to capture context-rich real-world insights into dietary behaviors, increase compliance, reduce missing data, improve data quality and accuracy, and reduce costs, participant burden, and recall bias [26,31,35,36]. Mobile apps could, thereby, generate large amounts of rich data that offer a detailed description of situational dietary behaviors [26,31] over longer time periods with relative ease [34]. Ecological momentary assessment (EMA) methods leverage the capabilities of mobile apps while enabling the assessment of dietary behaviors in real-world settings [37].

Notwithstanding these promising aspects, few studies to date have used mobile apps to explore dietary behaviors considering situational variances over a longer period of time [36]. Given the objectives of this study and the promising aspects of mobile apps and EMA for exploring real-world dietary behaviors, a mobile app was used to repeatedly and comprehensively sample situational snacking behaviors. This study, called FOODLOOP, used the mobile app Traqq (Traqq Pty Ltd) with EMA to support the detailed exploration of longitudinal situational snacking behaviors of a cohort of Dutch millennials. Specifically, FOODLOOP captured how real-world snacking behaviors vary within and across snacking situations given a comprehensive combination of product-, context-, and consumer-specific determinants. Even though the FOODLOOP study is descriptive in nature, it was guided by the expectations that (1) Dutch millennials would snack frequently; (2) snacking would show substantial variability both across and within a wide range of contexts, including locations, social settings, the time of day, days of the week, and the 4 seasons in a year; and (3) snacking would differ based on key consumer-specific characteristics.

## Methods

### Operationalization of Concepts

Given the different ways of defining snacking-related concepts in literature and the challenges this brings in unambiguously interpreting and comparing findings concerning snacking, the main snacking-related concepts as studied in FOODLOOP are operationalized as follows:

- Snack*:* a food or beverage product consumed outside the 3 traditional main meals (breakfast, lunch, and dinner)
- Snacking*:* the consumption of foods and/or beverages outside the 3 traditional main meals
- Snacking situation: the combination of a snack; FCMs for consuming a snack; and a physical, social, and temporal snacking context
- Snacking behavior: the totality of snacking situations of all participants

### Study Design

**Figure 1.**
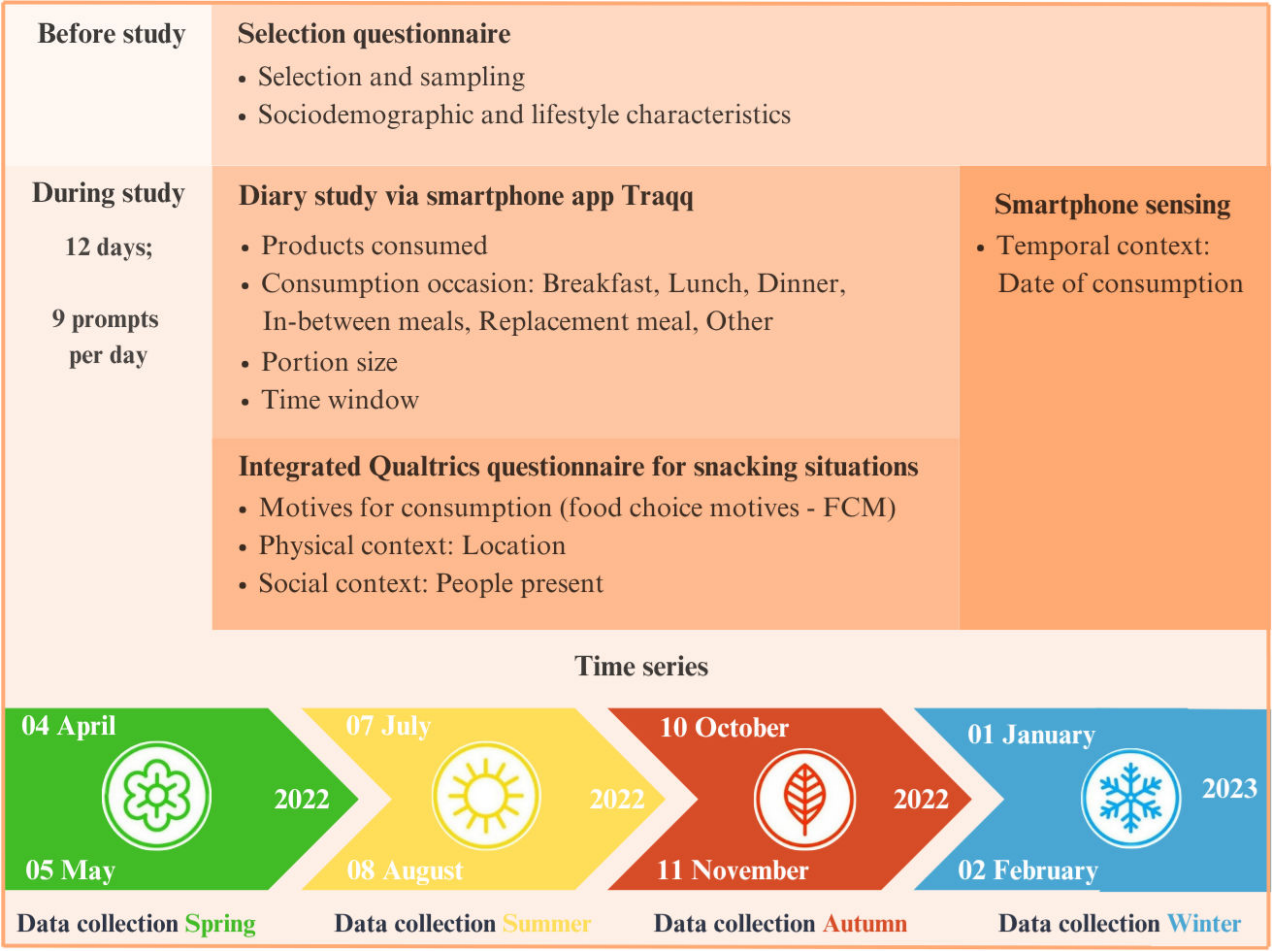
FOODLOOP data collection overview and time series structure.

The FOODLOOP study was carried out on a sample of 264 millennials (born between 1980 and 2000) residing in the Netherlands. Digital dietary diaries in a mobile app, following the principles of signal-contingent active EMA [33,37,38] and a time series structure [39], were used to collect data on participants’ self-reported snacking situations and situational variations. A selection questionnaire was used to gather potential participants, as well as data on participants’ sociodemographic and lifestyle characteristics (see Figure 1 for an overview of the study design).

Potential participants filled in the selection questionnaire. Once admitted to the study, participants participated in the diary study for 12 days, equally distributed over 4 data collection periods within 1 year (start: April 2022, end: February 2023). Following a time series structure (Figure 1), the 4 data collection periods took place over the course of 2 months every season. The data collection periods were separated by 1 month in which no data collection took place. In each data collection period, participants registered their data on 3 days to capture behavioral cycles [40]. The data collection days were randomly assigned to participants by an automatic sampling scheme per data collection period, for which a set of rules was written. These rules entailed that data collection days (1) were nonconsecutive; (2) consisted of 2 weekdays and 1 weekend day; and (3) took place within the 2 months deemed most representative for a particular season (Figure 1), which were chosen based on meteorological variations in different seasons in the Netherlands [41]. Participants had to successfully complete 3 data collection days within each data collection period, thus 12 data collection days in total. Participants were not informed about the dates of their data collection days beforehand. The whole FOODLOOP study, including the diary study and selection questionnaire, was conducted online.

### Ethical Considerations

The Social Sciences Ethical Committee of Wageningen University (WUR) approved consent procedures and study protocols of the FOODLOOP study (2022‐32-Vaal). The study was performed in accordance with ethical standards as laid down in Dutch privacy legislation and a data confidentiality declaration governed by Dutch law. To ensure participant privacy and confidentiality, all personal identifying information (such as names and contact information) was removed from the dataset before analysis, and participants were given a neutral identifier based on their study identifier. The research team only had access to the pseudoanonymized data. All participants provided informed consent to have their pseudoanonymized data published in journals. The compensation for participation in FOODLOOP consisted of a fixed monetary compensation, a lottery, and a donation possibility to heighten compliance [42-44]. Only participants who successfully completed all 12 data collection days received compensation. The fixed compensation consisted of €50 (US $59). Participants who successfully completed the study also automatically participated in a lottery where 10 prizes of €500 (US $585) were raffled. Participants received their compensation and prize after completion of the last data collection period (April 2023). Participants were given the option to keep the fixed compensation and prize themselves or have the research team donate the compensation and/or (part of) the prize on their behalf to the charitable organization Voedselbanken Nederland (Food Bank Netherlands), which provides basic food products to people who live below the poverty line. In total, €1800 (US $2106) was donated to Voedselbanken Nederland on behalf of the FOODLOOP cohort.

### Participant Selection and Compliance

Potential FOODLOOP participants were recruited via online platforms (social media and forums) and the snowball sampling technique [45]. Potential participants (n=798) filled in the selection questionnaire to screen them for their eligibility for participation in the follow-up diary study whilst collecting relevant data on sociodemographic and lifestyle characteristics (age, sex, education, urbanity, living situation, activity level, etc). Results were screened based on selection criteria, being that participants were (1) born between 1980 and 2000; (2) living in the Netherlands and Dutch-speaking; (3) eating and drinking outside of what they defined as main meals (ie, snacking); (4) not following a diet for medical reasons or under supervision of a health professional; and (5) not having eating disorders (eg, anorexia or bulimia). Based on the screening, 534 participants were eligible for participation in FOODLOOP and were invited for the diary study. In total, 398 participants consented to participation and started the diary study.

### Diary Study

#### Traqq

The FOODLOOP diary study was executed using the mobile app Traqq. Traqq (available for Android and iOS) was developed for dietary research purposes by Wageningen University [46] and allows for descriptive data collection, has no commercial focus, ensures privacy and safe storage of users’ data, and enables customizations in line with this study’s objectives. In Traqq, participants registered their daily food and beverage intake by choosing what they consumed from the Traqq food database, which includes products from the NEVO (Nederlands Voedingsstoffenbestand) 2016/2019 Dutch Food Composition Database [47,48] and Dutch food stores’ assortments. Additional variables measured for consumed snacks were captured through questionnaires in Qualtrics XM (2022‐2023). These questionnaires were programmed such that they appeared integrated within Traqq itself to participants. At the start of the study, participants received log-in credentials for Traqq, which remained valid throughout the entire study period. Once logged in, participants stayed continuously logged in, enabling them to receive prompts for all data collection days. Access to Traqq was deactivated upon study completion. All additions to and changes in features in the existing Traqq app to align it with FOODLOOP study objectives can be found in Table S1 in Multimedia Appendix 1.

#### Data Reporting Protocols

##### Signal-Contingent Active EMA

Given the nature of the study, the possibilities of the app, and the interference of the app with daily activities and social life when collecting data in real-time [49], it was decided to use signal-contingent active EMA for data collection. EMA allows for repeated data collection on participants’ situational dietary behaviors in their typical real-world context via mobile devices [37,38]. In the case of FOODLOOP, data were repeatedly collected on participants’ self-reported near real-time situational snacking behaviors in the physical, social, and temporal context in which they prevailed, via participants’ own mobile phones to reduce recall and social desirability bias.

Data collection days lasted 24 hours (06:00 AM-06:00 AM) and consisted of 9 prompts delivered via Traqq following a fixed schedule (Table S2 in Multimedia Appendix 1). Each prompt concerned one of the time windows and appeared as a push notification on the participant’s phone, which stayed salient for 2 hours until responded to. Participants indicated whether they had consumed anything during each time window. Nonresponses were registered as missing, and data collection days with too many missing prompts were considered invalid. Participants with invalid days were automatically rescheduled to repeat the session 2 days later within the designated data collection period until 3 valid days were achieved per data collection period.

##### Data Recording

During each of the 9 daily prompts, participants entered all food and beverage products consumed within the corresponding time window. They selected products from the Traqq database via the search bar and indicated the self-perceived portion size (Figure S1 in Multimedia Appendix 1). If a product was not listed, participants chose the most similar option available. Participants were instructed to record all consumed products except for plain water, fats and oils (eg, butter, margarine, and baking oil), and herbs and spices (Table S3 in Multimedia Appendix 1).

After entering the consumed product, participants had to label the product according to their perception of the associated consumption occasion as either one of the meal occasions (breakfast, lunch, and dinner) or one of the snacking occasions (replacement of meals, in-between-meals, or other), without giving instances of what foods and beverages constituted these occasions. This approach, adopted from Biel et al [36], allowed participants to self-define consumption occasions. Products of all consumption occasions, including meals, were recorded. Participants were not informed that FOODLOOP’s focus was on snacking behavior until after study completion to prevent bias among participants toward snacking entries.

##### Additional Questions for Snacks

**Figure 2.**
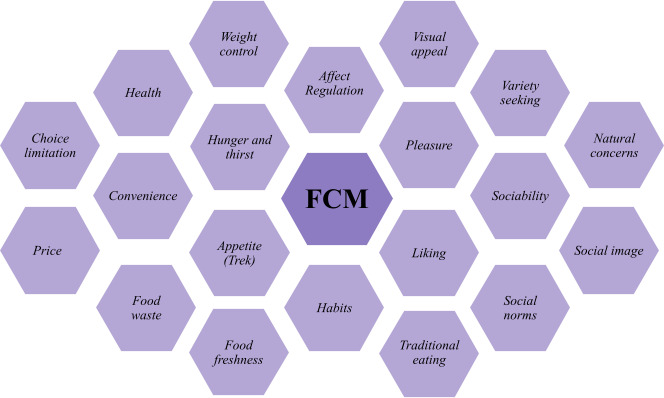
Food choice motive (FCM) of Dutch 1-scale-per-item Eating Motivation Survey (TEMS). FCM: food choice motive.

After entering a product and labeling the associated consumption occasion, participants received additional questions only when the product was marked as one of the snacking occasions. First, participants reported their FCM using the Dutch 1-scale-per-item Eating Motivation Survey (TEMS). This version contained 20 items (Figure 2), each assessing a unique FCM on a 3-point Likert scale ranging from 1 (“not applicable”) to 3 (“applicable”). The survey was based on the 1-scale-per-item TEMS used in the study of Wahl et al [26] and TEMS as used in multiple studies [27,30,50].

**Figure 3.**
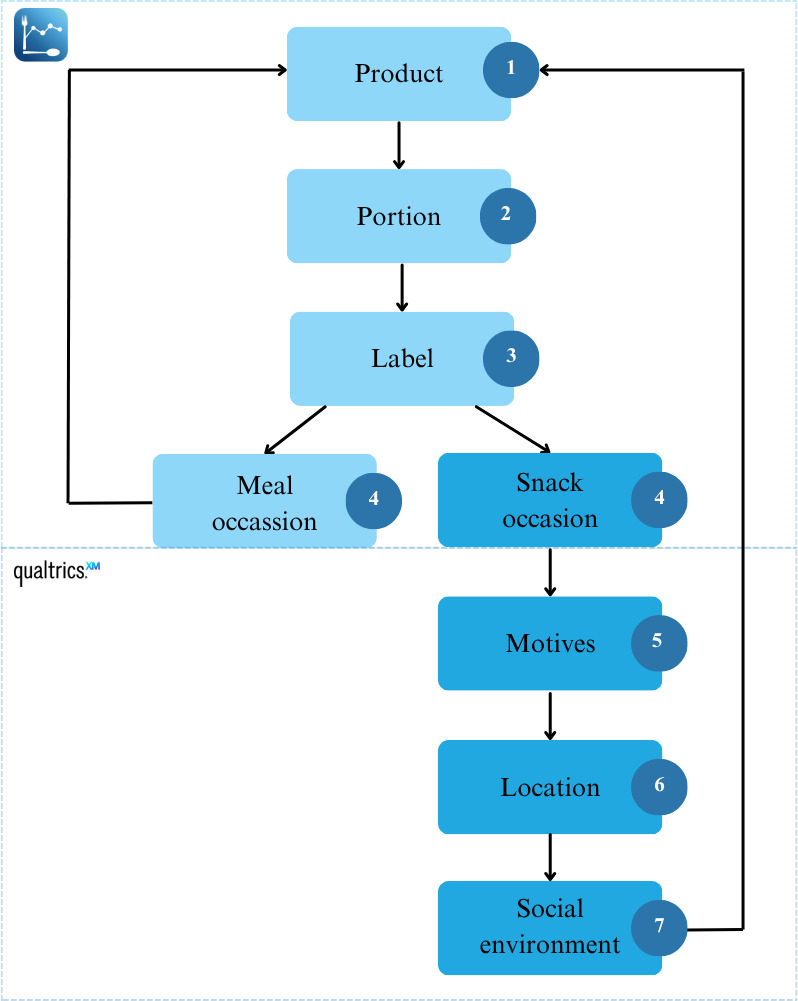
Protocol Traqq for entry of products.

Thereafter, participants indicated the physical (location) and social (people present) context of each consumed snack from predefined categories. The temporal context (eg, time window, type of day, and season) was derived after the study from the app’s recorded date and time data of each entry. When a product was labeled as a meal occasion or after completing a snacking entry, the app automatically returned to the Traqq home screen, where participants could log additional products or finalize the time window (Figure 3).

All consumed products were entered separately, with associated questions answered for each product individually (eg, if coffee and a cookie were consumed together, first the coffee was entered and questions about the coffee were answered, then the cookie was entered and questions about the cookie were answered). When participants entered all the products consumed within a time window and filled in the associated questions, or when participants indicated that they had not consumed anything, they completed the time window by submitting their answers. The time window was then recorded as successful. Data collection days with the required number of valid time windows were deemed successful, and participants remained promptable for subsequent data collection days. The app was refined through several pilot iterations to balance user burden and compliance. One data collection day took approximately 30 minutes to complete.

### Data Analysis

#### Data Preprocessing

Given the objectives of this study, only data concerning products labeled as snacking occasions were included in the analyses. Data related to meals, incomplete collection days, and dropout participants were excluded. Therefore, there were no participants with missing data for any of the variables of interest (Table 1). After data collection, all snack products were classified into categories (Table 1; Table S4 in Multimedia Appendix 1), based on the Dutch Food Composition Database (versions 2016 and 2019; NEVO [47,48]), Dutch National Food Consumption Survey [51,52], and the Netherlands Nutrition Center (Voedingscentrum [53]). These snack product category variables were subsequently used in the analyses. In addition, the variables concerning physical context were merged into 2 categories, “At home“ and “Out of home,” and the social context variables into the categories “Alone” and “With others.” Table 1 shows the snacking determinants, corresponding variables, and response categories for analyses.

**Table 1.** Snacking determinants, corresponding variables, and response categories for analyses.

Snacking determinants and variables		Response categories
Product		
Snack categories beverages (3)		Nonalcoholic beverages; alcoholic beverages; dairy (substitutes)
Snack categories foods (7)		Breads and cereals; fruits; vegetables and legumes; dairy (substitutes); meat (substitutes); cookies, bars, and pastries; confectionery and ice cream; savory snacks
Context		
Physical context (2)		At home; out of home
Social context (2)		Alone; with others
Temporal context		
Daypart (3)		Morning (06:00 AM-12:00 PM); afternoon (12:00 PM-06:00 PM); evening and night (06:00 PM-06:00 AM)
Type of day (2)		Weekday (Monday-Friday); Weekend day (Saturday and Sunday)
Season (4)		Spring, summer, autumn, and winter
Consumer		
Motives (FCM^a^; 20)		Liking; appetite; hunger and thirst; convenience; pleasure; health; visual appeal; habit; food freshness; sociability; variety seeking; weight concerns; choice limitations; price; food waste; traditional eating; social image; social norms; and natural concerns
Sociodemographic and lifestyles		
Age (2)		Younger millennials (1990‐2000); older millennials (1980‐1990)
Sex (2)		Men; women
Education (2)		Lower educated; higher educated
Net income per month (4)		Below modal (<€2000; <US $2340); modal (€2000–€3000; US $2340-US $3510); above modal (>€3000; >US $3510) not disclosed
Daily occupation (4)		None; study; paid job; study and paid job
Urbanity (5)		Rural; low urbanity; moderate urbanity; high urbanity; very high urbanity [54]
Living situation (2)		Living alone; living with others
Parental status (2)		No child or children; child or children
Grocery shopping autonomy (3)		No or little autonomy; shared autonomy; most or all autonomy
Diet (2)		No diet; diet
BMI group (4)		Underweight (<18.5); healthy weight (18.5‐25); overweight (25-30); obese (>30)
Activity level (3)		Nonactive; semiactive; norm-active [55]
Fit level (3)		Nonfit; semifit; norm-fit [55]
Health interest (1-7)		Very low health interest – … - very high health interests

a FCM: food choice motive.

All analyses were performed with standardized snacking frequencies for each response category of all considered variables (Table 1) to correct for unequal participant weights, as some participants recorded more snacks than others. Each snack originally represented one case in the dataset. To ensure that all participants contributed equally to the analyses, absolute snacking frequencies for each response category were divided by the number of data collection days (12), resulting in the daily average frequency (DAF) of snacks consumed. The DAFs provided a uniform measure of comparison, with each row in the dataset representing one participant rather than one snack. To account for the differing number of data collection days across contexts, the DAFs were adjusted for the number of days per week (for “Type of day”) and per season (for “Season”). DAFs were calculated for all response categories of all variables (Table 1) separately for (1) beverages as snacks, (2) foods as snacks, and (3) all snacks combined (for the detailed calculation of DAFs and all DAFs for each of the response categories, consult the Data Preprocessing and Descriptive Data sections and Tables S5-S8 in Multimedia Appendix 1).

#### Data Analyses

Data analyses were performed with Jamovi (Version 2.5; The jamovi Project), using R statistical packages (Version 4.1; R Core Team). Descriptive statistics and statistical tests were used to examine relationships between variables. In all analyses, a consumed snack represented a case (n) and the total number of participants was treated as the population (N). Descriptive statistics for snack category variables (Table S5 in Multimedia Appendix 1) demonstrated skewness toward nonalcoholic beverages (n=4204), indicating an imbalance between snack food and beverage categories. Given this imbalance and sufficient sample sizes for both beverages as snacks (n=5164) and foods as snacks (n=9148), analyses were conducted separately for these 2 groups.

Statistical tests were performed using DAFs of each response category (Table 1) to examine differences in snacking frequencies across determinants. Each variable was analyzed individually, aligning with the study’s aim to explore snacking determinants one-dimensionally, as these determinants were not mutually exclusive (eg, the same snack could be categorized as “At home,” “Alone,” and “In the morning”). For each variable, differences in DAFs were tested both between and within response categories for (1) beverages as snacks, (2) foods as snacks, and (3) all snacks combined (eg, comparing DAFs of “Alone” vs “With others” and subsequently beverages vs foods within each category).

Nonparametric tests were used given nonnormality (Shapiro-Wilk; *P*<.05). No sensitivity analyses or multivariable confounder-adjusted models were used, given the descriptive nature of the study. Differences in DAFs for product-, context-, and motivation-related variables were analyzed using Friedman ANOVA with Durbin-Conover pairwise comparisons. For motivation variables, McDonald ω was calculated to assess reliability [56,57], showing high internal consistency across all 20 FCM scales (beverages: *ω*=0.81; foods: *ω*=0.82; all snacks: *ω*=0.84). Subgroup analyses were conducted to explore differences in DAFs across sociodemographic and lifestyle variables and were analyzed using 2-tailed independent samples *t* tests, Mann-Whitney *U*, and Kruskal-Wallis tests. Depending on Levene test results, either Student *t* (*P*>.05) or Mann-Whitney *U* (*P*≤.05) was used for 2-category variables, while Kruskal-Wallis tests (Shapiro-Wilk, *P*≤.05) with Dwass-Steel-Critchlow-Fligner pairwise comparisons were applied for variables with more than 2 categories. Test values (except *P* values) were reported with 2 decimals, due to which minor rounding deviations may occur. For all statistical tests, a *P* value of ≤.05 was considered significant.

## Results

### Sample Compliance and Characteristics

Of the 398 participants who started the study, 264 (66.3%) fulfilled the entire year of data collection. These 264 Dutch millennials constitute the cohort of this study, the FOODLOOP cohort. More details about compliance rates throughout the longitudinal study period can be found in Table S9 and Figure S2 in Multimedia Appendix 1. Most of the FOODLOOP cohort consisted of women (232/264, 87.8%), who were highly educated (224/264, 84.8%), had a paid job (216/264, 81.8%), and a relatively high income (>€3000; US $3510) net income per month; 136/264, 51.5%). The ages of the participants ranged from 22 to 42 years (mean 30.14, SD 5.63) at the start of the study, with 72.3% (191/264) of participants belonging to the younger millennial age group (born between 1990 and 2000). As for the living situation of the participants, most were living in more urbanized areas of the Netherlands (198/264, 75%), with others (216/264, 81.2%), especially with a partner (108/264, 40.9%), and did not have children (212/264, 80.3%). Most participants could make grocery shopping decisions autonomously (158/264, 59.8%) or share this decision with other members in the household (100/264, 37.8%).

Seventy-two percent of participants (n=190) had a healthy weight (BMI: mean 23.28, SD 3.92) and 59.8% (158/264) did not follow a particular diet. Also, 21.2% (56/264) of participants skipped at least 1 meal a day or replaced meals with smaller consumptions. Most participants engaged in moderate and/or intense exercise at least once a week (moderate: 260/264, 98.5%; intense: 209/264, 79.1%), of which 39.3% (104/264) engaged in moderate exercise more than 5 times a week, and 26.5% (70/264) engaged in intense exercise more than 3 times a week. Weighted health interest of participants ranged from 2.75 to 6.88, with a mean of 5.02 (SD 0.78), indicating higher interest in health amongst participants in the FOODLOOP cohort.

### Product-Specific Determinants

**Figure 4.**
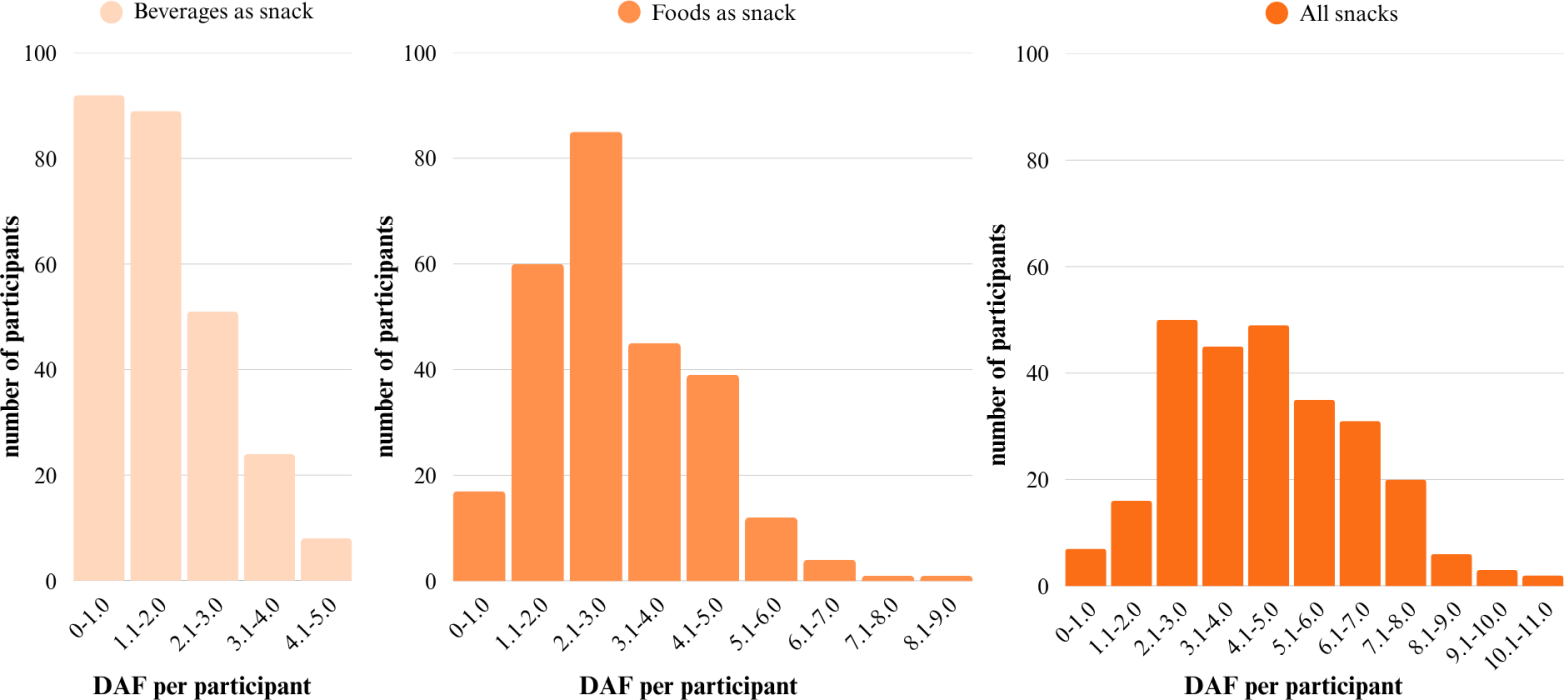
Distribution daily average frequencies (DAFs) of all participants for beverages as snacks, foods as snacks, and all snacks.

Over the longitudinal study period, 14,312 snacks, of which 5164 beverages as snacks (36.1%) and 9148 snack foods (63.9%), were consumed by the 264 participants in the FOODLOOP cohort over the course of 2987 unique days, across 4 different seasons. The DAFs of snacking range from 0 to 5 for beverages, 0 to 9 for foods, and 0 to 11 for all snacks. Most participants consumed 1‐2 beverages, 1‐4 foods, and 1‐5 snacks in total per day (Figure 4). On average, 1.63 beverages, 2.89 foods, and 4.52 snacks in total were consumed per participant per day. In all snacking situations, DAFs of foods were significantly higher than those of beverages (Table S10, Multimedia Appendix 1).

### Snacking Categories

**Figure 5.**
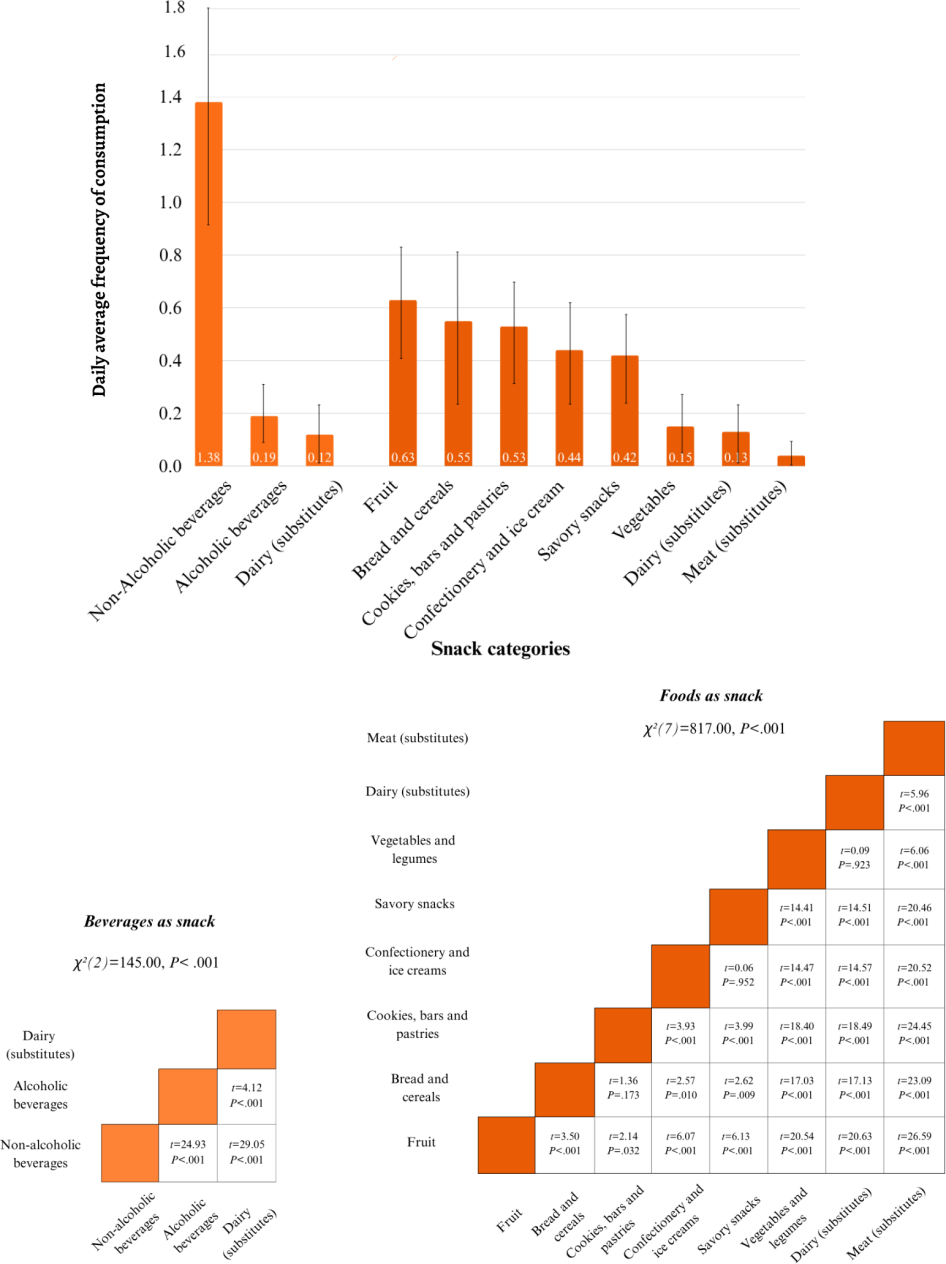
Daily average frequencies (DAFs) for snack categories and pairwise comparisons of differences between snack categories. Higher *t* values imply larger differences between DAFs of 2 snack categories.

Nonalcoholic beverages (consisting of mainly coffee and tea) were mostly consumed as snacks. For beverage categories, this was followed by alcoholic beverages. Dairy beverages and substitutes were least consumed. All differences between DAFs of the beverage categories were statistically significant (Figure 5).

Of all foods, fruit was mostly consumed as snacks, followed by bread and cereals; cookies, bars, and pastries; confectionery and ice cream; and savory snacks. Meat, poultry, fish, eggs, and substitutes were the least consumed foods as snacks. Most differences between DAFs for foods as snack categories were statistically significant (Figure 5). Most consumed snacks were perceived as an in-between meal consumption occasion. For replacement meal occasions, the DAF of foods was higher than that of beverages, whereas the DAF of foods was lower than that of beverages for snacking occasions other than in-between or replacement meals. All differences were statistically significant (Tables S10 and S11 in Multimedia Appendix 1).

### Context-Specific Snacking Determinants

#### Physical Context of Snacking

**Figure 6.**
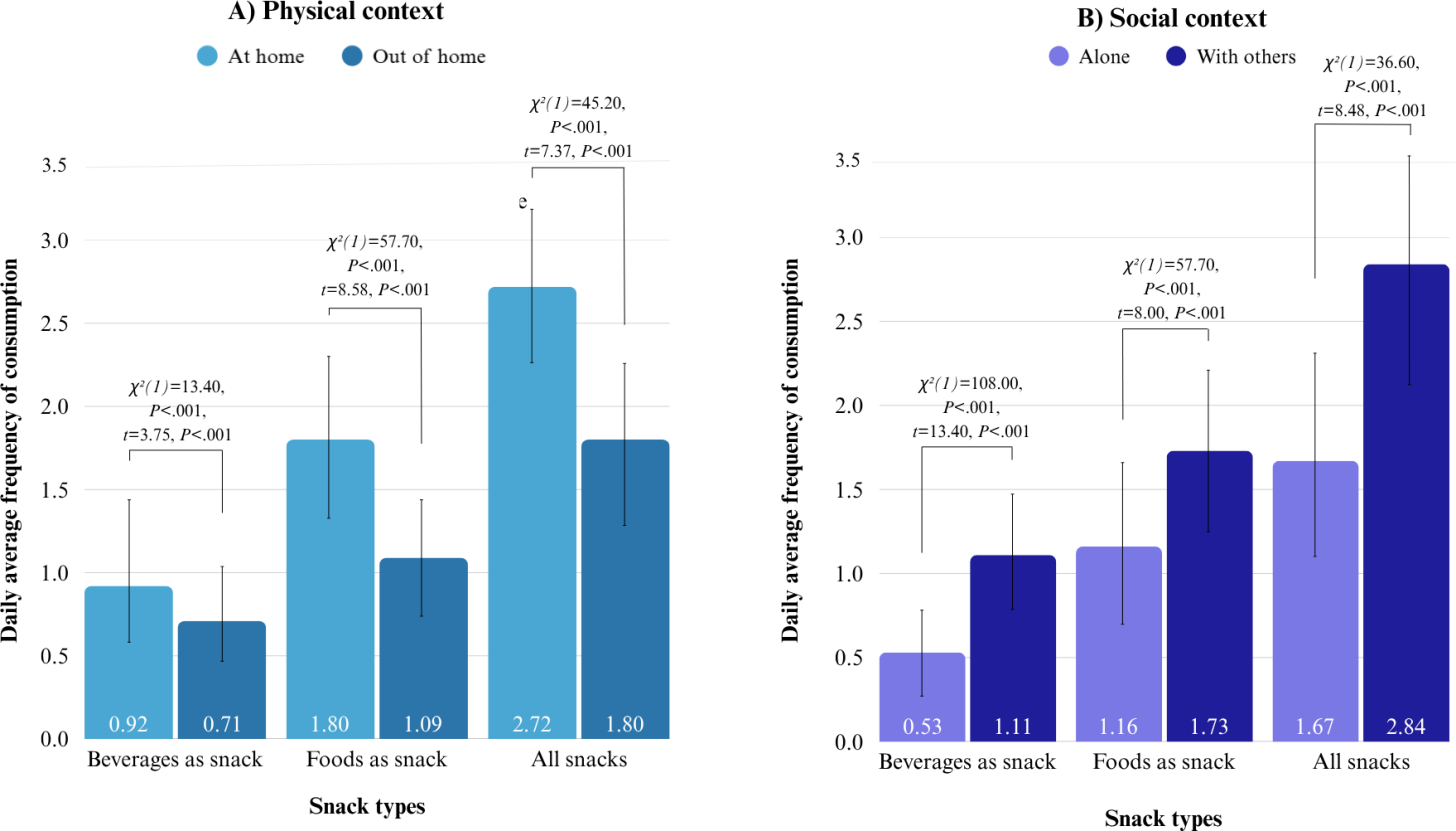
Bar charts of daily average frequencies (DAFs) of consumption and pairwise comparisons showing differences between DAFs in the (A) physical context at home versus out of home and (B) social context alone versus with others. Higher chi-square values imply larger differences between DAFs of 2 context categories.

Most snacks were consumed at home rather than out of home. This was the case for both foods and beverages (Figure 6A). In both contexts, the DAFs of foods were higher than those of beverages, but at home, the difference was larger. This indicates that foods were relatively more frequently consumed than beverages at home compared to out of the home. All differences were statistically significant (Figure 6A; Table S10 in Multimedia Appendix 1).

#### Social Context of Snacking

Most foods and beverages as snacks were consumed with others, rather than alone (Figure 6B). In both contexts, the DAFs of foods were higher than those of beverages. However, this difference was larger when alone. This indicates that foods were relatively more frequently consumed than beverages alone, as compared to others. All differences were statistically significant (Figure 6B; Table S10 in Multimedia Appendix 1).

#### Temporal Context of Snacking

##### Snacking During the Day

**Figure 7.**
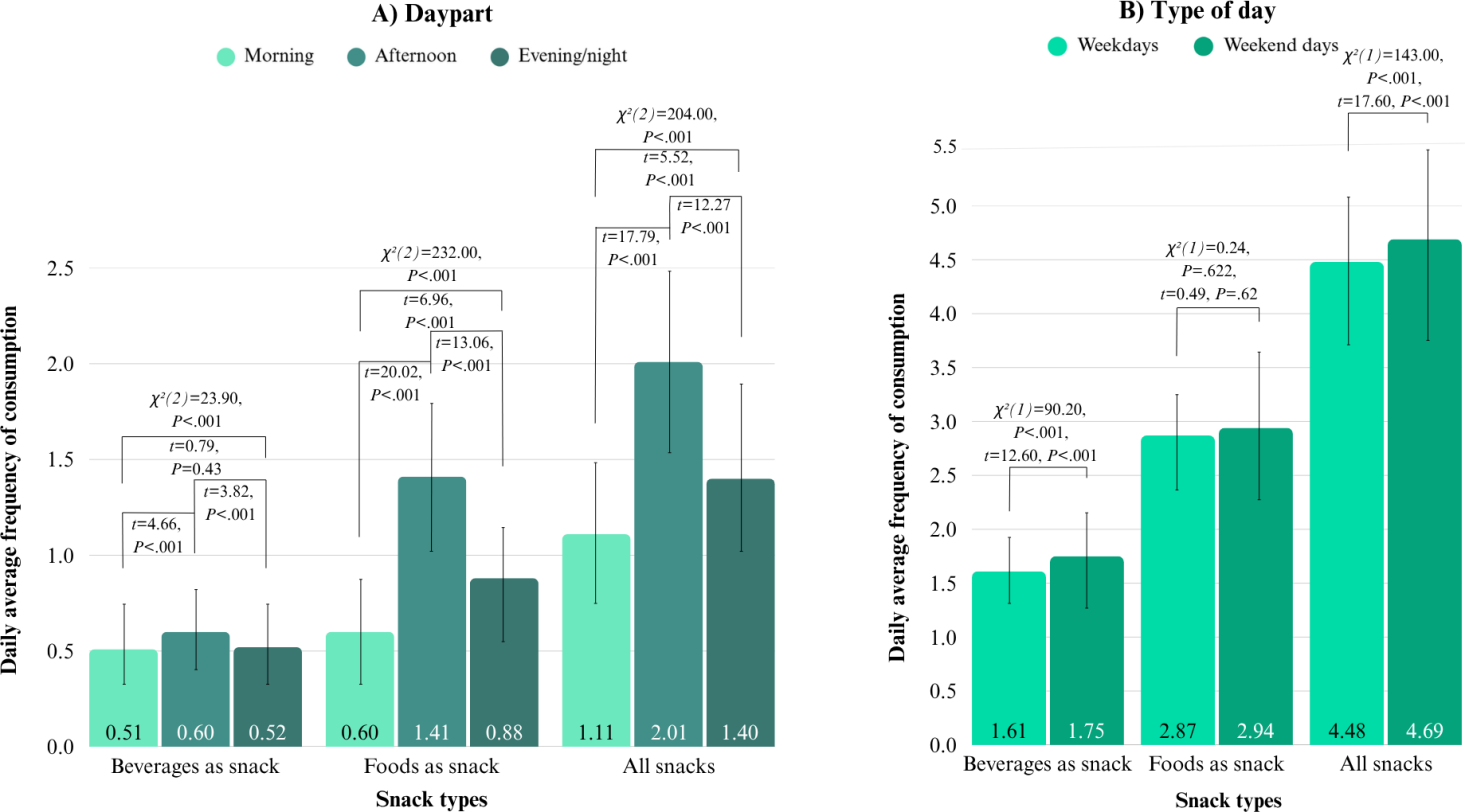
Bar charts of daily average frequencies (DAFs) of consumption and pairwise comparisons showing differences between DAFs in the temporal contexts. (A) Daypart, morning versus afternoon versus evening and night, and (B) day types, weekdays versus weekend days. Higher chi-square values imply larger differences between DAFs of 2 context categories.

Data demonstrate that snacks were consumed throughout the day, but most snacks were consumed in the afternoon, followed by the evening and night, and least snacks were consumed in the morning. This was the case for both foods and beverages but especially pronounced for foods. For foods, the DAF was lowest in the morning, whereas the DAFs of beverages did not differ significantly between the morning and the evening and night (Figure 7A). In all dayparts, DAFs of foods were significantly higher than those of beverages, but this difference was largest in the afternoon and smallest in the morning. This indicates that foods were relatively more frequently consumed than beverages in the afternoon as compared to other dayparts (Figure 7A; Table S10 in Multimedia Appendix 1).

##### Snacking During the Week

Data show that snack consumption on weekdays and weekend days was comparable. There were only minor differences in snack consumption between weekdays and weekend days. The weighted DAFs (accounting for the number of weekdays and weekend days) show that relatively more beverages were consumed on weekend days than on weekdays. Even though the difference was minimal, it was statistically significant. The weighted DAFs of foods did not differ significantly between weekdays and weekend days (Figure 7B). In both day types, the DAFs of foods were significantly higher than those of beverages, but this difference was larger on weekend days. This indicates that foods were relatively more frequently consumed than beverages on weekend days as compared to weekdays (Figure 7B; Table S10 in Multimedia Appendix 1).

##### Snacking During the Year

**Figure 8.**
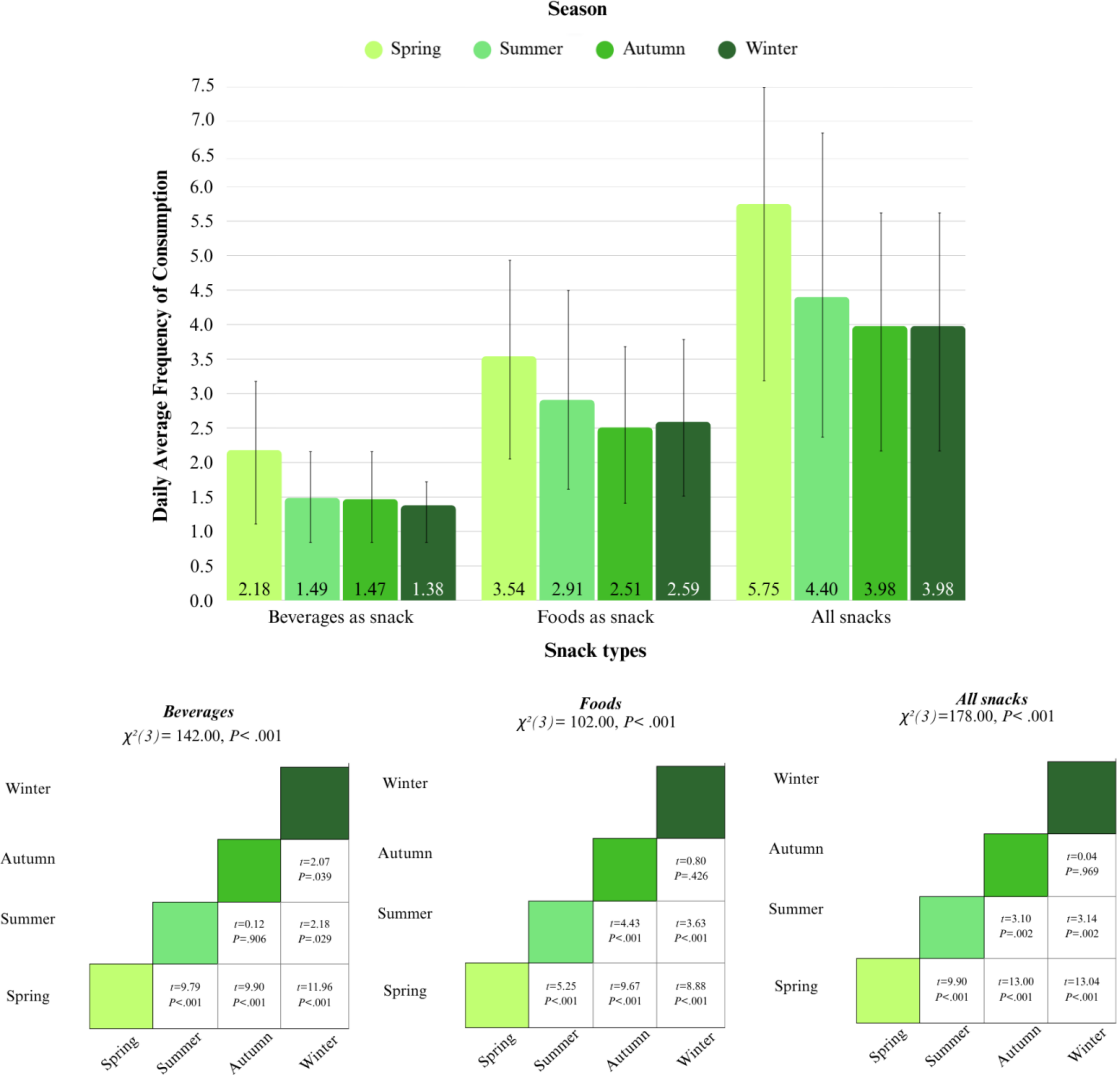
Bar charts for daily average frequencies (DAFs) of temporal context and pairwise comparisons. Seasonality of differences in DAFs between spring versus summer versus autumn versus winter. Higher chi-square values and *t* values imply larger differences between DAFs of seasons.

Most snacks were consumed in spring, followed by summer. DAFs of snacking were lowest in autumn and winter. Most differences in DAFs between seasons were significant. Only the DAFs of beverages did not differ significantly between summer and autumn, and the DAFs of foods did not differ significantly between autumn and winter (Figure 8). Within all seasons, the DAFs of foods were significantly higher than those of beverages. This difference was most pronounced in summer, indicating that foods were relatively more frequently consumed than beverages as compared to other seasons (Figure 8; Table S10, Multimedia Appendix 1).

### Consumer-Specific Snacking Determinants

#### Snacking FCMs

**Figure 9.**
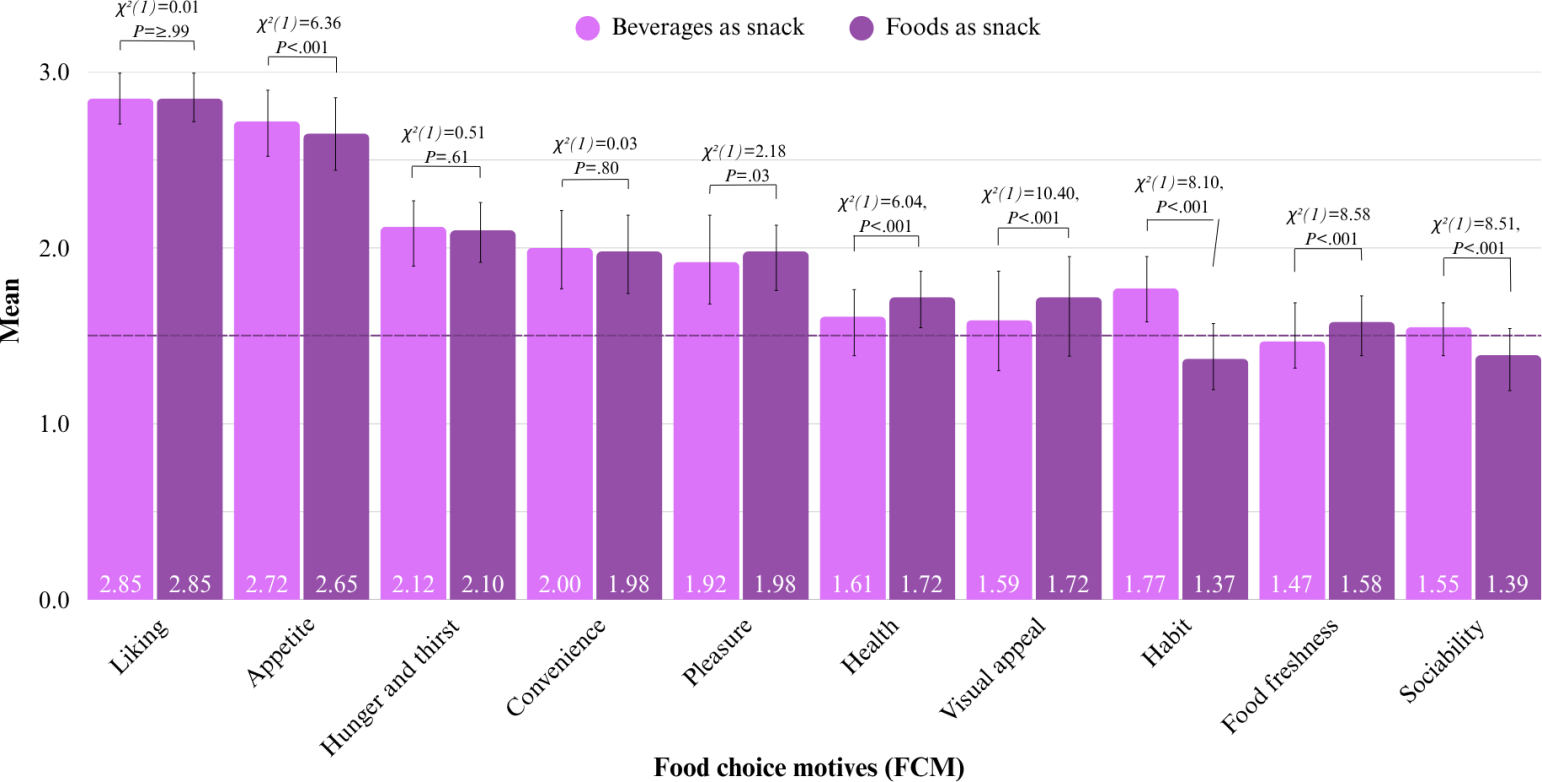
Average scores on food choice motives (FCM): pairwise comparisons results of FCM with means ≥1.5 (measured on 3-point Likert scales).

Data demonstrate that 10 out of the 20 FCMs played a prominent role in snacking (mean ≥1.5; Table S7 in Multimedia Appendix 1). Snacking was mainly motivated by liking and appetite (mean >2.5), followed by hunger and thirst, and convenience. Pleasure, visual appeal, health, habit, and food freshness were also found to belong to the core snacking drivers. The other 10 FCMs, including variety seeking, weight concerns, and affect regulation, did not often motivate participants to snack (mean <1.5). The role of most of the prominent FCM (mean ≥1.5) differed significantly between beverages and foods (Figure 9). These differences were largest for the FCM visual appeal, habit, and sociability. For beverages, the FCM habit, sociability, and appetite were more prominent, whereas visual appeal, health, pleasure, and food freshness were more prominent for foods. The other FCMs, liking, hunger and thirst, and convenience, were shown to be more universal snacking drivers, as they played a similar role in the consumption of both beverages and foods (Figure 9).

#### Sociodemographic, Lifestyle Factors, and Snacking

**Figure 10.**
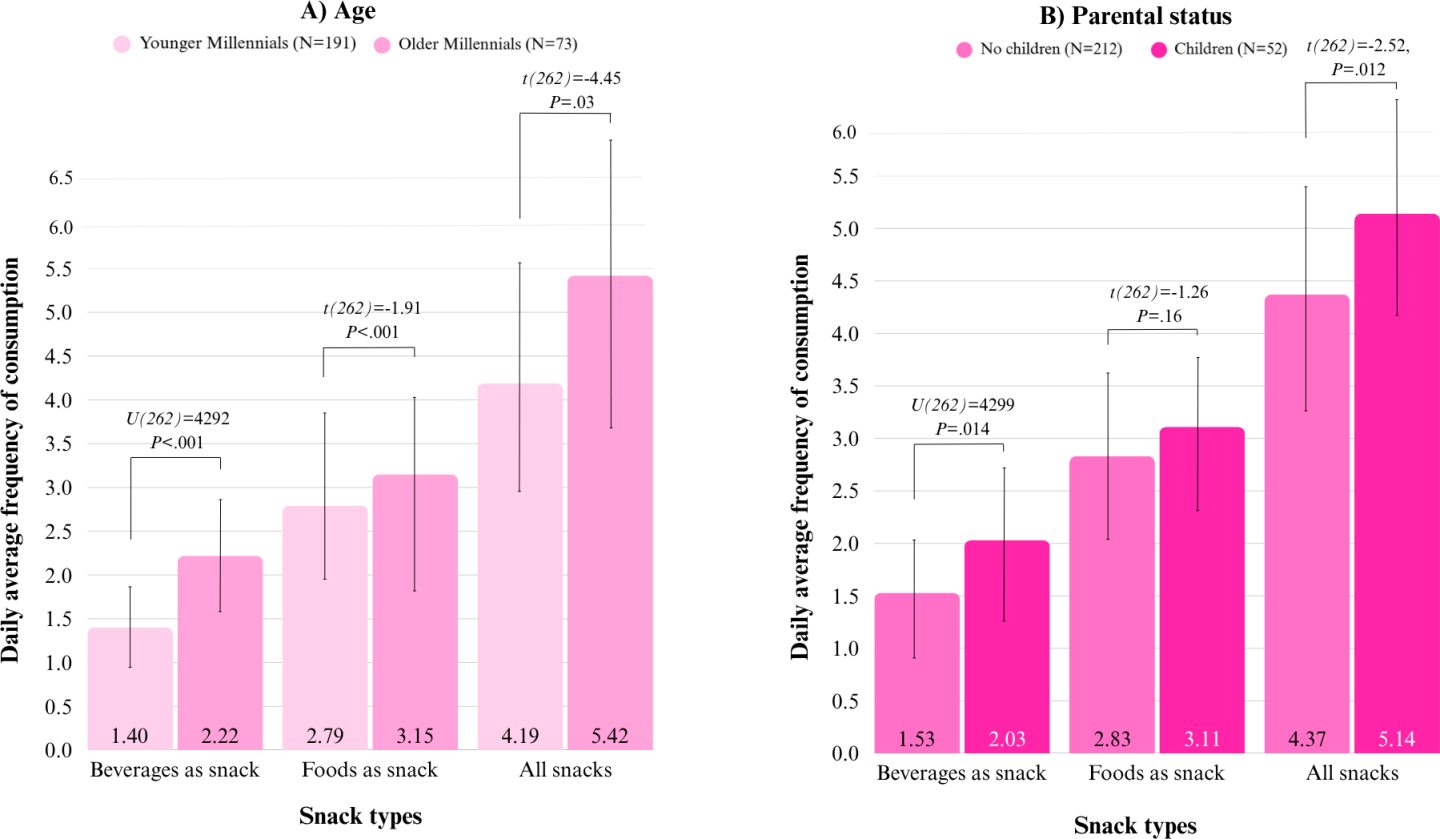
Bar chart showing daily average frequencies (DAFs) of consumption and pairwise comparisons showing differences between DAFs according to the sociodemographic characteristics of age and parental status. (A) Younger versus older millennials and (B) participants with children versus without children. Higher *U* values and *t* values imply larger differences between DAFs of demographic groups.

Regarding sociodemographic and lifestyle groups, the data demonstrate that there were statistically significant differences in DAFs for beverages and foods, given participants’ age and parental status. Participants belonging to the older millennials (born between 1980 and 1990) consumed more beverages and foods than participants belonging to the younger millennials (1990-2000) (Figure 10A). Participants with children consumed significantly more beverages than participants without children (Figure 10B). There were no statistically significant differences in DAFs for beverages and foods, given other sociodemographic and lifestyle factors of the participants, such as sex, education, urbanity of living area, and the activity and fit levels.

## Discussion

### Overview

This study used Dutch millennials as a case to explore consumers’ longitudinal situational snacking behaviors by investigating the role of product-, context-, and consumer-specific determinants in snacking situations, as well as how foods and beverages for snacking differ within and across these determinants, using a mobile app following the principles of signal-contingent active EMA.

### Principal Findings

#### Product-Specific Determinants

Examining the aggregated EMA data across all participants and snacking situations, this study found a daily average snacking frequency of 4.5. This is higher than reported in other studies, which range between 1.5 and 3.5 [58-62]. The higher frequency in our study might be explained by the composition of our cohort (1) millennials, who tend to snack more than other age groups, especially given the ongoing snackification trend [16], and (2) Dutch consumers, who likely adhere to the Dutch diet. As found by Geurts et al [14], Dutch adults (aged 19‐69 years) report more than 7 eating occasions per day, including 4 between-meal (ie, snacking) occasions, which aligns with our findings. Together, these findings suggest that snacking forms a substantial part of the daily diet of the Dutch, classifying them as heavy snackers (>3 snacks per day [21]).

Across all contexts, participants consumed relatively more foods than beverages as snacks. This contrasts with van Rossum et al [15], who found that the Dutch diet in general consists of more beverages than foods. However, our findings support existing snacking literature, showing that beverages are less prominent than foods in snack frequency, as beverages are less likely to be perceived as snacks by consumers [63,64].

Notwithstanding this smaller role of beverages in snacking, our findings do show that nonalcoholic beverages (mainly coffee and tea) were frequently consumed as snacks. This aligns with the Dutch diet in general being characterized by high consumption of nonalcoholic beverages [14,15] and the trend that an increasing proportion of beverages is consumed as snacks [21].

Concerning food categories, fruit, bread and cereals, cookies, bars and pastries, confectionery and ice creams, and savory snacks were among the top snacks for Dutch millennials. Vegetables, meat, and dairy (substitutes) were the least frequently consumed snacks. Most of these findings are consistent with previous studies [15,62,65,66]. Different from other studies is that our results show relatively high consumption of bread and cereal products as snacks. This may be explained by findings from other Dutch studies [14,15], showing that bread and cereal products are staple foods and important energy sources in the Dutch diet, especially for meal occasions. Our results suggest that these foods also play an important role in Dutch millennials’ snacking occasions.

#### Context-Specific Determinants

Confirming our expectations, findings show that context plays a central role in shaping snacking behaviors. Findings regarding the physical context show that most snacks were consumed at home, aligning with previous studies [29,67-69] and national dietary data showing that over 85% of foods and beverages in the Netherlands are consumed at home [14,15]. This could be explained by the convenience of the accessibility to snacks at home and the cost-effectiveness of home snacking [70]. The timing of data collection, shortly after the COVID-19 pandemic (2022‐2023), may also have influenced these results. Postpandemic patterns show that consumers not only snack more frequently in general [71-73], but especially at home, a habit established during lockdowns [74-76]. Furthermore, the COVID-19 pandemic remnant of remote and hybrid working [77] may have increased participants’ time spent at home, thereby facilitating more home snacking.

Concerning the social context, our study shows that most snacks were consumed with others, rather than alone. Since information regarding social snacking consumption patterns of the Dutch is scarce [14], our findings offer new insights into the role of the social environment in Dutch millennials’ snacking. Our findings, however, contrast with social snacking consumption patterns in other Western countries, where snacking is considered a personal eating event and mostly done alone [3,50,78,79]. A possible explanation for the higher consumption of snacks with others in our study might be the interaction between the physical and social environment; most snacks were consumed at home, and since most of our cohort lived with partners, children, and/or family members, snacking at home may have naturally occurred in the presence of other inhabitants.

Concerning the temporal context, our study shows that snacking occurred throughout the day. This suggests that snacking is not necessarily confined to intervals between the regular Dutch mealtimes, but that any consumption at any time not perceived as (part of) a meal can be a snack. This is in line with findings of other studies [3,80,81] and the snackification trend [82]. Throughout the day, snacking was most prevalent in the afternoon, followed by the evening and morning, consistent with prior studies [60,69,83]. To the best of our knowledge, our study is one of the first to explore seasonal variations in consumers’ snacking behaviors. We show that snack consumption was the highest in spring and the lowest in autumn and winter. These seasonal variations in snacking frequency might be explained by the seasonal climate of the Netherlands, where variations in meteorological factors such as temperature, precipitation, sunshine hours, and humidity are more noticeable throughout the seasons. In such climates, seasonal variations are also more pronounced in food consumption [84]. Even though literature on the effect of seasonality on food behaviors is scarce, our findings support those of Girju and Ratchford [85], who also observed less snacking in winter and more snacking in spring and summer.

#### Consumer-Specific Determinants

Concerning sociodemographic and lifestyle factors, we found some differences in snacking frequencies given age and parental status. Interestingly, older millennials snacked more frequently in general than younger millennials, and millennials with children consumed more beverages as snacks than those without. These 2 findings might be connected, as most of the participants with children were older millennials. Our findings concerning age align with those of Geurts et al [14], who reported that overall food consumption among the Dutch increases with age until middle age. The lack of expected differences in snacking frequencies for other sociodemographic and lifestyle factors, including sex, education, and activity levels, may reflect a possible type II error around the estimates, given minor subgroups of participants for factor levels [86].

Concerning FCMs, our study extends prior work by capturing within-person variations in snacking motives across different snacking contexts, thereby providing a deeper understanding of why consumers choose specific snacks in specific situations. We found that liking was the primary driver for snacking in all consumption situations, congruent with other studies [50,62]. Other core motivators for snacking in our study, including hunger and thirst, convenience, weight control, health, pleasure, and visual appeal, are also aligned with findings from other studies. Different from other studies is that we found other core FCM for snacking to entail sociability, food freshness, and habit [50,62]. The role of these FCMs in our study might be explained by the interaction of multiple snacking determinants, as the motives driving consumers’ snacking behaviors depend largely on the consumption context [29,50,87].

The role of FCM Sociability in our study could be explained by the finding that most snacks were consumed in the presence of others. The statement measuring this FCM included the Dutch word “gezellig.” The meaning of “gezellig” is not straightforward [88] but can be explained as the experience of togetherness in doing everyday activities together, often at specific times of the day and in one’s own home, implying collective enjoyment [88,89]. The “gezelligheid” of consuming snacks with others has played a noteworthy role in our study, possibly due to the social enjoyment and collectiveness of snacking. Even though “gezelligheid” is culturally embedded within the Dutch snacking context, the TEMS has demonstrated high cross-country and cultural group validity [26,30]. Therefore, our findings concerning FCM sociability might be translatable to other cultural contexts given sociability-related concepts similar to “gezelligheid,” but future studies are warranted.

The role of food freshness might be explained by our finding that mostly fruits, bread, and cereals were consumed as snacks. Food freshness includes the recency of production or harvest and the overall product quality [90-93]. It is a key determinant of consumers’ food choices, especially for fruits and baked products, which are highly susceptible to deterioration over time and perceived as having higher quality closer to the moment of harvest, production, or purchase [91,92]. This interaction of snack product categories and FCM might have played a role in our study.

The prominence of the motive habit in snacking has been established in other studies, but mainly for unhealthy snacking behaviors [94] and fruit intake in general [95]. The high consumption of fruit as a snack, alongside the cumulative consumption of cookies, bars, and pastries; savory snacks; and confectionery and ice creams in our study, might explain the prominent role of the FCM habit.

The prominence of the FCMs health and hunger and thirst in our study demonstrates that our cohort often selected snacks based on perceived healthfulness and satiating properties. Health- and hunger-driven snacking tends to be associated with the consumption of more healthful products [9,96]. This is observed in our study, as 64% of all consumed snacks consisted of coffee and tea, fruits, bread and cereal products, dairy products, and vegetables. This challenges the traditional view of snacking as a merely unhealthy dietary behavior characterized by underconsumption of nutrient- and fiber-rich products (such as fruits and grains) and excessive consumption of highly palatable traditional snack foods high in salt, saturated fat, trans fat, and sugar [97,98]. Our findings thus contrast with the traditional representation of snacking as merely unhealthy and harmful and support the growing scientific evidence that argues that snacking is not necessarily detrimental to but could also potentially benefit health [7,12,13,99]. We endorse the notion of Barnes et al [60] that snacking, like any other dietary behavior, can be practiced in a healthful or nonhealthful manner, depending on the product chosen, variety within the diet, and the underlying motives for consumption.

### Strengths

One of the main strengths of the FOODLOOP study is the use of a mobile app following a signal-contingent active EMA method, which enabled the collection of data on situational snacking behaviors in participants’ natural environments, using the routinized handling of mobile phones. Large amounts of detailed data concerning participants’ near real-time snacking behaviors, together with the comprehensive combination of product-, context-, and consumer-specific determinants present at specific moments in unique snacking situations, were documented, which allows for a better understanding of real-world snacking behaviors. As beverages consumed as snacks were distinctly captured, the data also enabled the analyses of differences in snacking behaviors between foods and beverages, accounting for variances in situational determinants. Given the use of a mobile app following an EMA method, practical applications of the findings from FOODLOOP are expected to be more relevant to real-world snacking situations [100] while minimizing missing data, memory loss, recall bias, and social desirability bias and maximizing ecological validity [26,31,35,36,38,46].

Another strength is the longitudinal nature of our study. FOODLOOP is one of the first to use a time-series structure for longitudinal data collection on consumers’ snacking behaviors, enabling the exploration of temporal relationships among variables, especially the little-studied seasonal variations in snacking. As data were collected from the same group of 264 participants in all data collection periods, both inter- and intraindividual variability in snacking behaviors could be analyzed. The completion rate of 66.3% (264 of 398 participants) is comparable to those of previous EMA studies [101,102], and the FOODLOOP study managed to keep compliance of the participants throughout several data collection periods throughout the year, notwithstanding months in which no data collection took place. Participants’ compliance was supported by the FOODLOOP study design in 3 ways:

- Limiting participant burden through the data collection structure
  - Data were collected on 3 days each season, as 3-day surveys are shown to capture data with similar sufficiency to surveys with more data collection days [40].
  - Prompts were sent as push notifications on participants’ phones to heighten engagement and minimize data loss [103].
  - No prompts were sent between 11:00 PM and 08:00 AM to ensure that participants were not bothered at inappropriate times.
  - The number of questions per prompt was minimized while capturing all necessary data (eg, through using the extensively piloted 1-scale-per-item TEMS).
  - Notifications were sent once per prompt to prevent notification excessiveness, a known barrier in mobile app-based dietary intake [33,103,104].
- Communication with participants
  - Participants received concise and detailed user manuals (documents and videos) to heighten compliance [103-106] via email and the study’s web page before data collection, which remained continuously accessible during the study.
  - Participants received personalized reminder emails after each data collection day about the progress of participation.
  - The research team remained available 24/7 to enhance participants’ motivation [83,105,107-110] via email and Instagram (Meta) for participants’ remarks and questions during the full length of the study, including the no-data-collection months.
  - Emails and direct messages from participants were responded to within a day.
- The incentive scheme
  - The combination of multiple types of incentives (fixed monetary incentive, lottery, and donation possibilities) is the most effective strategy for increasing compliance rates [42-44] in studies using dietary mobile apps [105,111].

### Limitations

Notwithstanding the insights we provide into Dutch millennial consumers’ situational snacking behaviors through longitudinal EMA time-series assessment, several limitations should be acknowledged.

First, the sample was skewed toward women and higher-educated millennials, and most participants were living in urbanized areas, with a healthy weight and more active lifestyles. This likely resulted from recruiting participants through health- and nutrition-related channels (eg, Netherlands Nutrition Center and nutrition blogs) using a snowball sampling technique, rather than targeted sampling. This selection bias could have caused the inclusion of participants with a stronger interest in health and nutrition than the average Dutch millennial, which might have influenced the findings. Other possible forms of selection bias in this study could be self-selection, as participants who partook were likely more interested or experienced in tracking dietary intake throughout the day. Finally, there may be nonresponse and dropout bias, as participants failing to complete the study were often less motivated or unable to adhere to the sampling scheme due to being in settings (eg, working in the hospital) where using a phone every 2 hours was infeasible. Even though the profile of the FOODLOOP participants is skewed toward higher-educated, urban, health-conscious, active women, therewith limiting the generalizability of our findings to the broader Dutch or millennial population, the profile of our participants aligns with the group most engaged in the snackification trend [112]. Future studies are needed to explore whether the findings can be replicated in more balanced samples by including more men, lower-educated people, people living in more rural areas, and those with lower health interests and activity levels.

Second, sociodemographic and lifestyle variables were measured once at the beginning of the study and were therefore treated as static features in our analyses. However, some of these variables are susceptible to change over time (change in weight, meal patterns and diets, household composition, daily occupation, etc). Such changes were not accounted for during the data collection and might have influenced outcomes concerning the relation between snacking frequencies and sociodemographic and lifestyle variables. Furthermore, residual confounding by unmeasured sociodemographic and lifestyle variables cannot be excluded. Future longitudinal studies exploring the relation of sociodemographic and lifestyle variables with snacking should measure these variables at multiple moments during the data collection to account for possible changes over time and to enable analyses of how changes in these variables affect snacking behaviors.

Third, our findings should also be interpreted in light of the broader postpandemic context in which they took place (2022‐2023). Even though data collection did not prevail during the COVID-19 pandemic, several behavioral changes that may have emerged during the pandemic (eg, remote and hybrid working, different daily routines, more time spent at home than before) could have persisted into FOODLOOP data collection periods. This might have altered participants’ snacking situations and partly explain some of our findings, such as more snacking in general, as well as more home-based and healthful snacking [71-77,113,114]. Future research could examine whether these snacking behaviors are short-term postpandemic adaptations or reflect more stable long-term snacking behavioral changes.

Finally, this study explored a selection of determinants in snacking situations, due to which the generalizability of our findings to other determinants is limited, similar to the study of Verain et al [29]. Capturing snacking situations in their entirety is challenging, given the number of determinants playing a role in real-world snacking situations and the complexity of the interrelatedness of these determinants. However, many of the main determinants in snacking situations [29,115,116] were included in our analyses. The determinants that were not measured (eg, satisfaction with snack choice, activities executed during snacking, and participant’s mood) might constitute interesting topics of exploration for future snacking-related studies.

### Implications and Conclusion

Despite the limitations, the FOODLOOP study offers empirical insights into Dutch millennial consumers’ real-world snacking behaviors and how multiple situational determinants relate to these behaviors over a longer time period. FOODLOOP shows that snacking is a multifaceted situational behavior, where a comprehensive combination of product-, context-, and consumer-specific determinants plays a role. Confirming the expectations guiding our work, snacking occurs frequently and varies both across and within contexts, including the 4 seasons. This suggests that snacking is not restricted to specific products, motives, locations, social settings, or times, including anything can be a snack, consumed for any reason, anywhere, with anyone, and at any time, congruent with the snackification trend.

The contributions of our findings to research are 2-fold. First, the substantive insights into situational variances in Dutch millennials’ real-world snacking behaviors extend prior work on snacking and highlight the importance of its multidimensional nature. Our findings encourage future work to explore situational snacking behaviors across other age groups and cultural contexts and to further investigate interactions between situational determinants, including FCMs, seasonality, and meteorological factors. Future studies may also build on our findings to explore consumers’ perceptions and definitions of snacking, factors influencing interindividual variations in definitions of snacking, and the role of beverages as standalone snacks. Second, the methodological experiences could inspire future research on consumers’ snacking and other dietary behaviors. FOODLOOP demonstrates the value of using a mobile app based on EMA to study longitudinal, real-world dietary behaviors. The data collection structure, app programming, measure design, and participant communication provide a methodological framework that can be adopted and further refined in future studies on situational snacking and other dietary behaviors.

Beyond research, our findings also have practical relevance. The real-world situational snacking insights gained in FOODLOOP through EMA could support health professionals, nutrition experts, and policymakers in designing more effective and realistic, tailored interventions (eg, campaigns, guidelines, and public health strategies) aimed at encouraging healthier snacking patterns by targeting specific snacking occasions (locations, social settings, moments, and motives) to intervene in the critical moments of situations that are associated with more snacking. Furthermore, our results could inform the development of mobile app–based dietary tools in more effectively guiding healthier snacking choices. For instance, our insights could be used to create context-sensitive features, such as timed prompts and personalized nudges providing healthier snacking options that are suitable for a given context (eg, home alone in the afternoon) and that match users’ snacking motives in such situations (eg, convenience, hunger and thirst, and liking).

Overall, the FOODLOOP study underscores the importance of considering snacking as multidimensional and situational behavior within the growing snackification trend. Our insights provide guidance for research and practice in developing more targeted, context-sensitive approaches and interventions that deepen the understanding of snacking and support healthier snacking behaviors.

## Supplementary material

10.2196/71858 Multimedia Appendix 1 Additional detailed information on methodological decisions (Traqq app changes; study measures) and data preprocessing, detailed descriptive statistics and statistical test results for the presented findings, and participant attrition details.

## Abbreviations

DAF: daily average frequency
EMA: ecological momentary assessment
FCM: food choice motive
TEMS: The Eating Motivation Survey
