# Supplementary material for: Exploring Consumers’ Situational Snacking Behaviors Using a Mobile App: Longitudinal Cohort Study (FOODLOOP) Among Millennials in the Netherlands
Source: JMIR Mhealth Uhealth. 2026 May 26;14:e71858. doi: 10.2196/71858 (PMC13204325; doi:10.2196/71858)
Supplement: Multimedia Appendix 1 [file mhealth-v14-e71858-s001.docx]

# Multimedia Appendix 1

***Manuscript title***: Exploring Consumers’ Situational Snacking Behaviors Using a Mobile App: FOODLOOP - a Longitudinal Cohort Study Among Millennials in the Netherlands

***Manuscript ID:*** 71858

### Methodology

**Table S1.** Additions and Changes Existing Traqq App for FOODLOOP study.

| **Features** | **Existing Traqq app** | **Additions and changes** |
| --- | --- | --- |
|  |  |  |
| Product database | NEVO 2016 [^48,49^]. | - NEVO 2019[^48,49^]: plant-based dairy and meat substitutes. - Frequently consumed products which are not present in NEVO 2016/2019, based on assortments in Dutch supermarkets (Albert Heijn, Jumbo, Plus, etc.) and on-the-go facilities (AH To Go, Kiosk, Shell Select, etc.):  Meal salads, Protein powder, Ready-made bread rolls, etc. |
| Portion of consumption | Participants manually choose exact weight of consumption in grams. | Participants chose regular serving sizes (e.g. tablespoon, cup, glass, bowl) with associated indication of weight, as well as the number of chosen serving sizes to estimate portions. |
| Time of consumption | Participant manually choose exact time of consumption (HH:MM). | Inference of time based on time windows and mobile-sensed time of entry to lower participant burden. |
| Consumption occasion labels | *Breakfast; Lunch; Dinner; Tussendoor (=In-between).* | Addition of labels: *Replacement of breakfast*; *Replacement of lunch*; *Replacement of dinner*; *Other* to enable more thorough identification of different types of snacking occasions. |
| Motives and context | No features measuring consumption motives or context. | Integrated link to Qualtrics questionnaires concerning Food Choice Motives and contextual determinants for products labeled as one of the snacking occasions. |
| Diary schedule | 24-h diary recall or customizable EMA structure. | Signal-contingent active EMA schedule with 9 prompts:   - 8 prompts concerning 2-hour time windows: 06:00-08:00; 08:00-10:00; …; 20:00-22:00. - 1 prompt concerning an 8-hour time window: 22:00-06:00 to prevent disturbance during the night. |
| Sampling scheme | Customizable data collection days, salience of prompts, allowed non-response per day and rescheduling of incomplete data collection days. | Data collection days:   - Data collection on 3 days per data collection period, including 2 weekdays and 1 weekend day. - No 2 data collection days in a row.   Salience of prompts:   - Participants had to respond to prompts within 2 hours after the time window ended.   Non-response:   - Participants were allowed to miss the first (06:00-08:00), last (22:00-06:00) or one random prompt. - Invalid days were repeated 2 days after the initial day, until the data collection period ended or 3 valid days were completed. |

**Table S2.** Prompting schedule data collection day FOODLOOP.

| **Time window** | **Prompt receival** | **Respond before** | **Target snacking occasions** |
| --- | --- | --- | --- |
| 06:00-08:00 | 08:00 | 10:00 | Pre-breakfast, Replacement breakfast |
| 08:00-10:00 | 10:00 | 12:00 | Coffee-time, After breakfast, Replacement breakfast, Pre-lunch |
| 10:00-12:00 | 12:00 | 14:00 | Coffee-time, Replacement breakfast, Mid-morning In-between, Pre-lunch, Replacement lunch |
| 12:00-14:00 | 14:00 | 16:00 | Pre-lunch, After lunch, Replacement lunch, Early afternoon In-between |
| 14:00-16:00 | 16:00 | 18:00 | After lunch, Replacement lunch, Mid/Late afternoon In-between, Pre-dinner, Replacement dinner |
| 16:00-18:00 | 18:00 | 20:00 | Late afternoon In-between, Pre-dinner, Replacement, Dinner, After dinner |
| 18:00–20:00 | 20:00 | 22:00 | Replacement dinner, After dinner, Pre-bed |
| 20:00-22:00 | 22:00 | 00:00 | After dinner, Pre-bed, Late-night In-between |
|  | The next day: |  |  |
| 22:00-06:00 | 08:00 | 10:00 | Late-night In-between, Nighttime In-between, pre-breakfast, replacement breakfast |


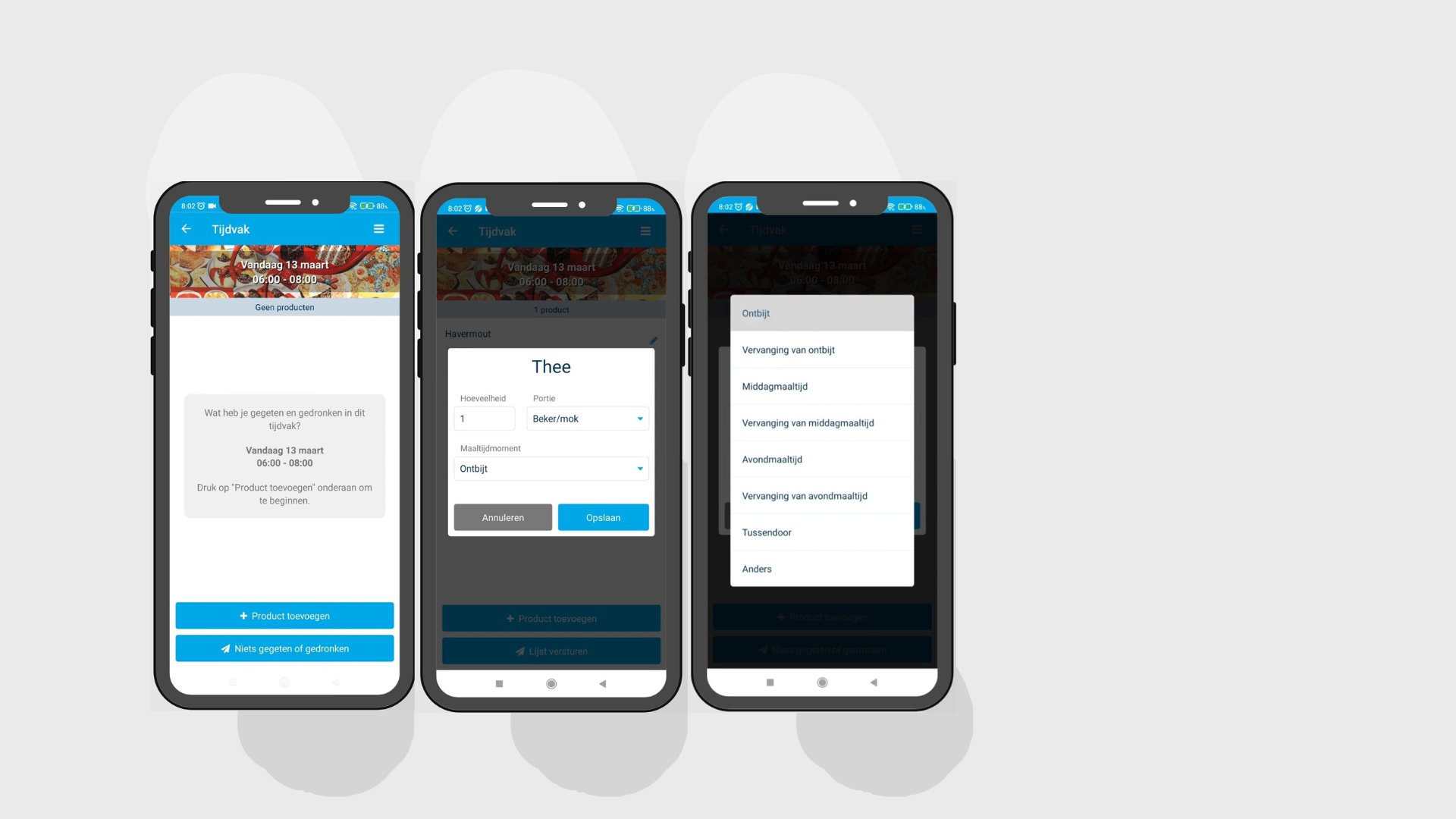


**Figure S1.** Traqq interface for reporting products: left, opening screen for time window; middle, window for indicating a product and product quantity; right, window for indicating a consumption occasion.

**Table S3.** Non-documented products FOODLOOP study.

| **Products to not document** | **Examples** | **Rationale** |
| --- | --- | --- |
|  |  |  |
| Plain water | Tap water, non-carbonated mineral water | - *Reduce participant’s burden*: Having to indicate every sip of water, added spice/herb, used oils/fat separately would heighten the burden of participation. - *Prevent lack and invariability of reporting:* Occasions when participants might consume water, fats and oils, herbs and spices, but might not register given the inherent use. - *Prevent inconsistent analyses of results:* Applying the researchers definitions of products for the analyses, whereas participants’ definitions might differ. - *Inflated consumption rates:* Each reported sip of water, added herb/spice, fat/oil would greatly heighten consumption rates. - *Relative importance for understanding snacking behaviour:* As the products are additions to other snacking products, but not the core of the consumption, the relative importance for snacking is lower.   (Biel et al., 2018; Chan et al., 2021; Gage et al., 2021; Wahl et al., 2020). |
| Fats and oils | Butter, margarine, oil, baking spray |  |
| Herbs and spices | Flavour enhancers, e.g. Cinnamon, parsley, spice mix. |  |

#### Data pre-processing

**Snack Categories**

**Table S4** . Snack product categories used in analyses.

| **Snack product categories** | **Description and examples** |
| --- | --- |
|  |  |
| **Beverages as snack** |  |
|  |  |
| Non-alcoholic beverages | Coffee and tea, carbonated water, fruit drinks/juices, and (sugar-free) soft drinks. |
| Alcoholic beverages | Beer and wine, cocktails, liquors, and spirits. |
| Dairy (substitutes) | Milk products, (protein) shakes, smoothies, and plant-based substitutes (e.g. almond, soy, and oat drinks). |
|  |  |
| **Foods as snack** |  |
|  |  |
| Bread and cereals | Bread rolls, wraps and sandwiches (ready-made), cornflakes, oatmeal,  crackers and toast (with toppings), and pancakes. |
| Fruit | Fresh and dried fruits. |
| Vegetables and legumes | Baked, cooked and raw vegetables and legumes. |
| Dairy (substitutes) | Custard, quark, yogurt and plant-based substitutes (e.g. almond, soy, and oat yoghurt/curd). |
| Meat (substitutes) | Baked, cooked and raw meat/chicken/fish/eggs and processed meats /fish/eggs (e.g. sausages, herring, boiled eggs) or meat/fish substitutes. |
| Cookies, bars and pastries | Biscuits, cakes, (cereal) bars, pies, sweet (puff) pastries. |
| Confectionery and ice cream | Candy, chocolate, ice cream, sorbet, sweet sauces. |
| Savory snacks | Cheese cubes, crisps, deep-fried snacks (e.g. frikandel, kroket), fries, nuts, popcorn, ready-made soups, saltines, savory (puff) pastries. |

**Calculation of Daily Average Frequency (DAF) of Snack Consumption**
Because participants reported varying numbers of snacks, absolute frequencies were standardized to ensure equal weighting across individuals. Each snack entry initially represented one case in the dataset. For each participant, the total number of snacks within a given response category (e.g., “Social context: Alone”) was divided by the total number of data collection days (12) to yield a *Daily Average Frequenc* (DAF).

To correct for the number of days represented by specific variables, the DAF was multiplied by:

- 7 for the variable “Type of day” (reflecting 7 days per week), and
- 4 for the variable “Season” (reflecting 4 seasons).

These adjusted DAFs provided a uniform, relative measure for comparison across participants. DAFs were calculated separately for:

1. Beverages consumed as snacks,
2. Foods consumed as snacks, and
3. All snacks combined.

***Example:***
For instance, if participant 1 consumed a total of 48 snacks over 12 days—comprising 18 beverages and 30 foods in the social context *“Alone”*—this resulted in:

- Total snacks: 48 ÷ 12 = 4 DAF alone
- Beverages as snack: 18 ÷ 12 = 1.5 DAF alone
- Foods as snack: 30 ÷ 12 = 2.5 DAF alone

After this standardization, each row in the dataset represented one participant rather than one snack, ensuring consistent weighting across all analyses.

### Results

#### Descriptive results

**Table S5.** Product-Specific Determinants Descriptives: Cases (n) and Daily Average Frequencies (DAFs) for Snack Categories (listed in order of consumption) and Perceived Snacking occasions.

| **Product-specific determinants** | | **n (%)** | **M (SD)** | **m (IQR)** |
| --- | --- | --- | --- | --- |
|  | |  |  |  |
| **Snack categories** | |  |  |  |
|  | |  |  |  |
| ***Beverages as snack (total)*** | | 5164 (36.1%) | 1.63 (1.33) | 1.25 (1.50) |
|  | Non-alcoholic beverages | 4204 (29.4%) | 1.38 (0.94) | 1.08 (1.27) |
|  | Alcoholic beverages | 590 (4.1%) | 0.19 (0.24) | 0.08 (0.25) |
|  | Dairy (substitutes) | 370 (2.6%) | 0.12 (0.22) | 0.00 (0.22) |
|  | |  |  |  |
| ***Foods as snack (total)*** | | 9148 (63.9%) | 2.89 (1.86) | 2.59 (2.16) |
|  | Fruit | 1988 (13.9%) | 0.63 (0.46) | 0.50 (0.60) |
|  | Bread and cereals | 1746 (12.2%) | 0.55 (0.53) | 0.42 (0.58) |
|  | Cookies, bars and pastries | 1691 (11.8%) | 0.53 (0.38) | 0.50 (0.50) |
|  | Confectionery and ice cream | 1383 (9.7%) | 0.44 (0.37) | 0.33 (0.42) |
|  | Savory snacks | 1313 (9.2%) | 0.42 (0.33) | 0.33 (0.42) |
|  | Vegetables and legumes | 466 (3.3%) | 0.15 (0.23) | 0.08 (0.19) |
|  | Dairy (substitutes) | 426 (3%) | 0.13 (0.27) | 0.08 (0.25) |
|  | Meat (substitutes) | 135 (0.9%) | 0.04 (0.10) | 0.00 (0.08) |
|  | |  |  |  |
|  | |  |  |  |
| **Perceived snacking occasions** | |  |  |  |
|  | |  |  |  |
| ***In-between meals*** | |  |  |  |
|  | Beverages as snack | 4669 (32.6%) | 1.48 (1.01) | 1.25 (1.42) |
|  | Foods as snack | 8197 (57.3%) | 2.59 (1.37) | 2.33 (1.92) |
|  | All snacks | 12866 (89.9%) | 4.06 (1.99) | 3.83 (2.94) |
|  | |  |  |  |
| ***Replacement meal*** | |  |  |  |
|  | Beverages as snack | 157 (1.1%) | 0.05 (0.10) | 0.00 (0.08) |
|  | Foods as snack | 722 (5 %) | 0.23 (0.41) | 0.08 (0.25) |
|  | All snacks | 879 (6.2%) | 0.16 (0.26) | 0.05 (0.19) |
|  | |  |  |  |
| ***Other*** *^a^* | |  |  |  |
|  | Beverages as snack | 338 (2.4 %) | 0.11 (0.30) | 0.00 (0.08) |
|  | Foods as snack | 229 (1.6%) | 0.07 (0.20) | 0.00 (0.08) |
|  | All snacks | 567 (4%) | 0.18 (0.45) | 0.00 (0.17) |

^a^*Other= Consumption occasions outside of the 3 main meals, which were not perceived as in-between or replacement meal occasion by participants.*

**Table S6.** Context-Specific Determinants Descriptives: Cases (n) and Daily Average Frequencies (DAFs) for Physical Context, Social Context and Temporal Context (Daypart, Day Type and Season).

| **Context-specific determinants** | | **n (%)** | **M (SD)** | **m (IQR)** |
| --- | --- | --- | --- | --- |
|  | |  |  |  |
| **Physical context** | |  |  |  |
|  | |  |  |  |
| ***At home*** | |  |  |  |
|  | 2916 (20.4%) | 2916 (20.4%) | 0.92 (0.79) | 0.67 (0.83) |
|  | 5694 (39.8%) | 5694 (39.8%) | 1.80 (1.11) | 1.67 (1.33) |
|  | 8610 (60.2%) | 8610 (60.2%) | 2.72 (1.63) | 2.38 (2.10) |
|  | |  |  |  |
| ***Out of home*** | |  |  |  |
|  | Beverages as snack | 2248 (15.7%) | 0.71 (0.53) | 0.58 (0.67) |
|  | Foods as snack | 3454 (24.2%) | 1.09 (0.74) | 0.92 (0.83) |
|  | All snacks | 5702 (39.8%) | 1.80 (1.08) | 1.58 (1.42) |
|  | |  |  |  |
|  | |  |  |  |
| **Social Context** | |  |  |  |
|  | |  |  |  |
| ***Alone*** | |  |  |  |
|  | Beverages as snack | 1663 (11.6%) | 0.53 (0.49) | 0.42 (0.58) |
|  | Foods as snack | 3677 (26%) | 1.16 (0.87) | 1.00 (1.00) |
|  | All snacks | 5340 (37.3%) | 1.69 (1.14) | 1.42 (1.35) |
|  | |  |  |  |
| ***With others*** | |  |  |  |
|  | Beverages as snack | 3501 (24.5%) | 1.11 (0.77) | 1.00 (1.08) |
|  | Foods as snack | 5471 (38.4%) | 1.73 (0.97) | 1.58 (1.35) |
|  | All snacks | 8972 (62.7%) | 2.84 (1.49) | 2.75 (2.19) |
|  | |  |  |  |
|  | |  |  |  |
| **Temporal context** | |  |  |  |
|  | |  |  |  |
| **Daypart** | |  |  |  |
|  | |  |  |  |
| ***Morning*** | |  |  |  |
|  | |  |  |  |
|  | Beverages as snack | 1604 (11.2%) | 0.51 (0.43) | 0.42 (0.58) |
|  | Foods as snack | 1906 (13.3%) | 0.60 (0.47) | 0.50 (0.58) |
|  | All snacks | 3510 (24.5%) | 1.11 (0.74) | 1.00 (1.00) |
|  | |  |  |  |
| ***Afternoon*** | |  |  |  |
|  | Beverages as snack | 1908 (13.3%) | 0.60 (0.42) | 0.50 (0.58) |
|  | Foods as snack | 4462 (31.2%) | 1.41 (0.72) | 1.33 (1.00) |
|  | All snacks | 6370 (44.5%) | 2.01 (0.94) | 1.92 (1.25) |
|  | |  |  |  |
| ***Evening/Night*** | |  |  |  |
|  | Beverages as snack | 1652 (11.5%) | 0.52 (0.41) | 0.50 (0.52) |
|  | Foods as snack | 2780 (19.4%) | 0.88 (0.64) | 0.75 (0.75) |
|  | All snacks | 4432 (31%) | 1.40 (0.84) | 1.25 (1.02) |
|  | |  |  |  |
|  | |  |  |  |
| **Day type** | |  |  |  |
|  | |  |  |  |
| ***Weekdays*** | |  |  |  |
|  | Beverages as snack | 3487 (24.4%) | 1.15 (0.80) | 0.95 (1.05) |
|  | Foods as snack | 6636 (46.8%) | 2.05 (1.05) | 1.90 (1.40) |
|  | All snacks | 10123 (70.7%) | 3.20 (1.50) | 3.00 (2.10) |
|  | |  |  |  |
| **Weekend days** | |  |  |  |
|  | Beverages as snack | 1577 (11%) | 0.50 (0.38) | 0.42 (0.50) |
|  | Foods as snack | 2612 (18.3%) | 0.84 (0.54) | 0.72 (0.72) |
|  | All snacks | 4189 (29.3%) | 1.34 (0.96) | 1.26 (1.22) |
|  | |  |  |  |
| ***Weekdays^b^*** | |  |  |  |
|  | Beverages as snack | 698 (4.9%) | 0.23 (0.16) | 0.19 (0.21) |
|  | Foods as snack | 1327 (9.3%) | 0.41 (0.21) | 0.38 (0.28) |
|  | All snacks | 2025 (14.1%) | 0.64 (0.30) | 0.60 (0.42) |
|  | |  |  |  |
| ***Weekend days^b^*** | |  |  |  |
|  | Beverages as snack | 788 (5.5%) | 0.25 (0.19) | 0.21 (0.25) |
|  | Foods as snack | 1306 (9.1%) | 0.42 (0.27) | 0.36 (0.38) |
|  | All snacks | 2094 (14.6%) | 0.67 (0.38) | 0.63 (0.54) |
|  | |  |  |  |
|  | |  |  |  |
| **Season** | |  |  |  |
|  | |  |  |  |
| **Spring** | |  |  |  |
|  | Beverages as snack | 1724 (12.1%) | 2.18 (2.00) | 1.28 (1.67) |
|  | Foods as snack | 2803 (19.6%) | 3.54 (3.33) | 1.89 (2.33) |
|  | All snacks | 4527 (31.6%) | 5.72 (5.33) | 2.50 (3.33) |
|  | |  |  |  |
| **Summer** | |  |  |  |
|  | Beverages as snack | 1180 (8.2%) | 1.49 (1.33) | 1.16 (1.33) |
|  | Foods as snack | 2302 (16.1%) | 2.91 (2.67) | 1.69 (2.33) |
|  | All snacks | 3482 (24.3%) | 4.40 (4.33) | 2.31 (3.00) |
|  | |  |  |  |
| **Autumn** | |  |  |  |
|  | Beverages as snack | 1164 (8.1%) | 1.47 (1.33) | 1.21 (1.33) |
|  | Foods as snack | 1988 (13.9%) | 2.51 (2.33) | 1.57 (2.00) |
|  | All snacks | 3152 (22%) | 3.98 (3.67) | 2.24 (3.00) |
|  | |  |  |  |
| **Winter** | |  |  |  |
|  | Beverages as snack | 1096 (7.7%) | 1.38 (1.00) | 1.24 (1.67) |
|  | Foods as snack | 2055 (14.4%) | 2.59 (2.33) | 1.63 (2.33) |
|  | All snacks | 3151 (22%) | 3.98 (3.67) | 2.29 (3.00) |
|  |  |  |  |  |

^b^ Weighted snacking frequencies: Weekday: …/5 ; Weekend day: …/2.

**Table S7.** Average scores Food Choice Motives (FCM), measured on a scale 1-3 with 1= not applicable; 2= somewhat applicable, and; 3= very applicable.

| **FCM** | **Beverages as snack** |  | **Foods as snack** |  | **All snacks** |  |
| --- | --- | --- | --- | --- | --- | --- |
|  | **M (SD)** | **m (IQR)** | **M (SD)** | **m (IQR)** | **M (SD)** | **m (IQR)** |
|  |  |  |  |  |  |  |
| Liking | 2.85 (0.20) | 2.93 (0.18) | 2.85 (0.17) | 2.90 (0.16) | 2.85 (0.18) | 2.91 (0.16) |
| Appetite | 2.72 (0.28) | 2.79 (0.30) | 2.65 (0.29) | 2.71 (0.34) | 2.69 (0.27) | 2.76 (0.32) |
| Hunger/thirst | 2.12 (0.43) | 2.14 (0.64) | 2.10 (0.40) | 2.12 (0.59) | 2.11 (0.38) | 2.13 (0.52) |
| Convenience | 2.00 (0.46) | 1.98 (0.73) | 1.98 (0.46) | 1.98 (0.72) | 1.99 (0.43) | 2.00 (0.66) |
| Pleasure | 1.92 (0.48) | 1.95 (0.73) | 1.98 (0.44) | 2.01 (0.65) | 1.95 (0.44) | 1.98 (0.67) |
| Health | 1.61 (0.40) | 1.54 (0.58) | 1.72 (0.36) | 1.67 (0.49) | 1.67 (0.34) | 1.63 (0.48) |
| Visual appeal | 1.59 (0.56) | 1.44 (0.76) | 1.72 (0.55) | 1.63 (0.87) | 1.66 (0.54) | 1.52 (0.78) |
| Habit | 1.77 (0.43) | 1.73 (0.63) | 1.37 (0.29) | 1.31 (0.38) | 1.57 (0.32) | 1.55 (0.42) |
| Food freshness | 1.47 (0.44) | 1.35 (0.62) | 1.58 (0.40) | 1.54 (0.60) | 1.53 (0.40) | 1.46 (0.58) |
| Sociability | 1.55 (0.38) | 1.48 (0.44) | 1.39 (0.30) | 1.33 (0.35) | 1.47 (0.31) | 1.43 (0.40) |
| Variety seeking | 1.38 (0.42) | 1.21 (0.52) | 1.35 (0.38) | 1.19 (0.53) | 1.37 (0.39) | 1.22 (0.53) |
| Weight concerns | 1.32 (0.37) | 1.19 (0.48) | 1.35 (0.38) | 1.25 (0.54) | 1.33 (0.38) | 1.23 (0.48) |
| Choice limitation | 1.19 (0.24) | 1.11 (0.23) | 1.29 (0.29) | 1.20 (0.34) | 1.24 (0.25) | 1.16 (0.27) |
| Affect regulation | 1.25 (0.32) | 1.13 (0.32) | 1.23 (0.31) | 1.10 (0.29) | 1.24 (0.30) | 1.12 (0.30) |
| Price | 1.18 (0.30) | 1.04 (0.24) | 1.17 (0.26) | 1.06 (0.23) | 1.18 (0.27) | 1.06 (0.24) |
| Food waste | 1.10 (0.15) | 1.05 (0.11) | 1.20 (0.21) | 1.13 (0.25) | 1.15 (0.17) | 1.09 (0.16) |
| Traditional eating | 1.15 (0.22) | 1.07 (0.19) | 1.12 (0.16) | 1.06 (0.18) | 1.13 (0.17) | 1.08 (0.16) |
| Social image | 1.14 (0.27) | 1.02 (0.14) | 1.11 (0.24) | 1.00 (0.12) | 1.13 (0.25) | 1.03 (0.12) |
| Social norms | 1.13 (0.18) | 1.06 (0.19) | 1.11 (0.16) | 1.05 (0.15) | 1.12 (0.17) | 1.06 (0.16) |
| Natural concerns | 1.04 (0.15) | 1.00 (0.04) | 1.04 (0.13) | 1.00 (0.03) | 1.04 (0.13) | 1.00 (0.04) |

**Table S8.** Socio-demographic and lifestyle factors descriptives: cases (n) and Daily Average Frequencies (DAFs) per factor.

| **Socio-demographic and lifestyle factors** | **n (%)** | **M (SD)** | **m (IQR)** |
| --- | --- | --- | --- |
|  |  |  |  |
| **Age group (N)** |  |  |  |
| **Younger Millennials (191)** |  |  |  |
| Beverages as snack | 3226 (22.5%) | 1.40 (0.87) | 1.25 (1.25) |
| Foods as snack | 6401 (44.7%) | 2.79 (1.35) | 2.50 (1.79) |
| All snacks | 9627 (67.3%) | 4.19 (1.89) | 4.00 (2.83) |
|  |  |  |  |
| **Older Millennials (73)** |  |  |  |
| Beverages as snack | 1938 (13.6%) | 2.22 (1.24) | 2.00 (2.00) |
| Foods as snack | 2747 (19.2%) | 3.15 (1.35) | 2.92 (1.92) |
| All snacks | 4685 (32.%) | 5.37 (2.03) | 5.42 (2.92) |
|  |  |  |  |
| **Parental status (N)** |  |  |  |
| **No children (212)** |  |  |  |
| Beverages as snack | 3901 (27.3%) | 1.53 (0.97) | 1.42 (1.42) |
| Foods as snack | 7208 (50.4%) | 2.83 (1.41) | 2.67 (1.83) |
| All snacks | 11109 (77.6%) | 4.37 (1.96) | 4.21 (3.08) |
|  |  |  |  |
| **Children (52)** |  |  |  |
| Beverages as snack | 1934 (13.5%) | 2.03 (1.26) | 1.83 (2.10) |
| Foods as snack | 1269 (8.9%) | 3.11 (1.31) | 2.96 (2.12) |
| All snacks | 3203 (22.4%) | 5.14 (2.07) | 4.79 (2.96) |

#### Attrition details


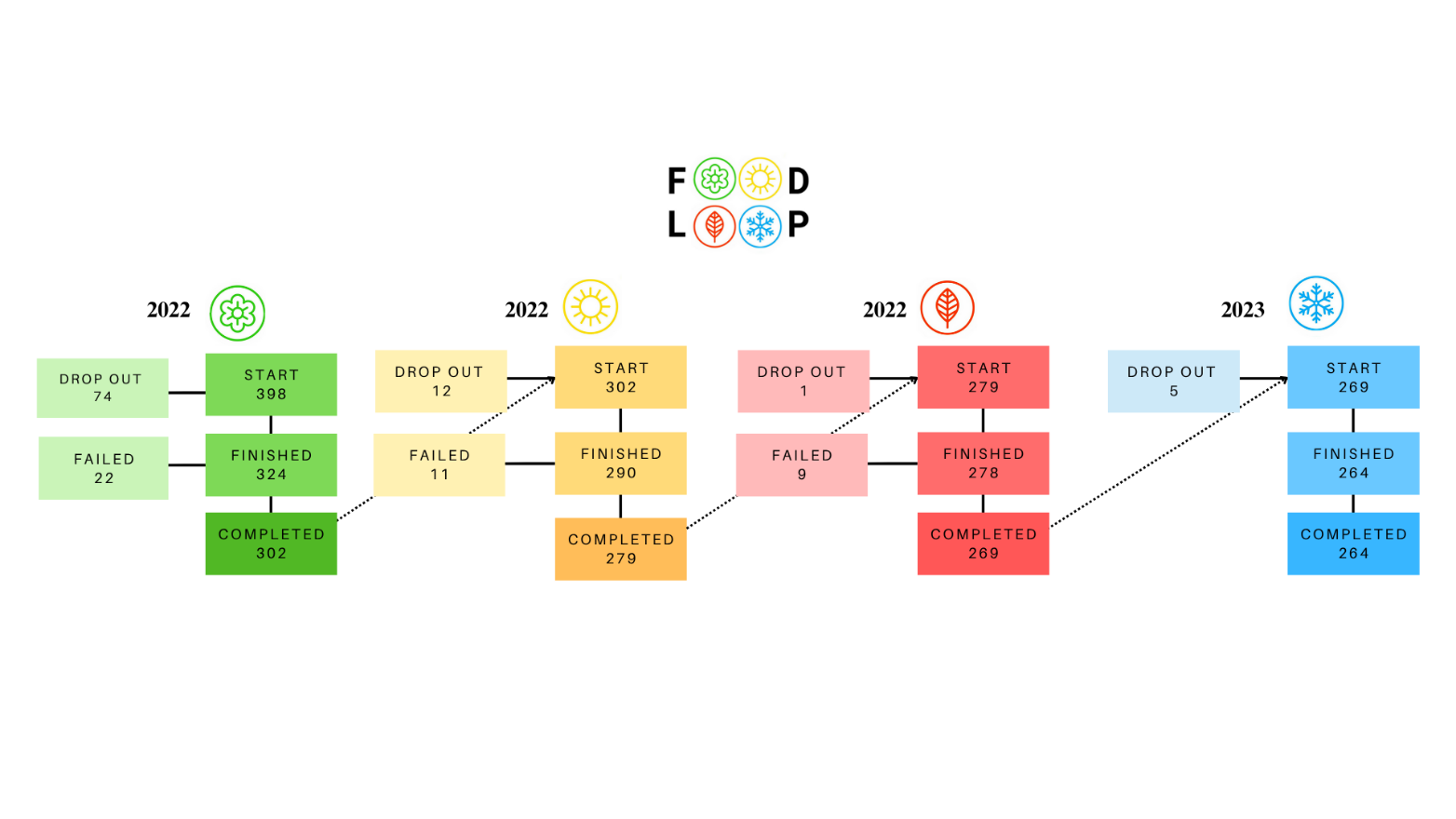


**Figure S2** . Flow diagram participant retention per data collection period.

Participant’s compliance per data collection period can be found in Figure S2 and Table S9. Most participants dropped out in the first data collection period due to burden of the study and not being able to respond to a sufficient number of prompts per day. After the first data collection period, dropout rates were minor and remained stable (Table S9).

**Table S9.** Compliance rates FOODLOOP participants per data collection period.

| **Data collection period** | **Started** N (%): | **Drop-out** N (%): | **Remained** N (%): |
| --- | --- | --- | --- |
|  |  |  |  |
| 1: Spring | 398  (100) | 96  (24.1) | 302  (75.9) |
| 2: Summer | 302  (100) | 23  (7.6) | 279  (92.4) |
| 3: Autumn | 279  (100) | 10  (3.6) | 269  (96.4) |
| 4: Winter | 269  (100) | 5  (1.9) | 264  (98.1) |
| Total drop-out N (%) /  remained N (%): 134 (33.7) / 264 (66.3) | | | |

#### Pairwise Comparisons

**Table S10.** Pairwise comparisons of Daily Average Frequencies (DAFs) for Beverages as Snack vs. Foods as Snack for Physical Context, Social Context, Temporal Context (Daypart, Day Type and Season) and Consumption occasion.

| **Comparison Beverages as Snack vs. Foods as Snack** | | **Friedman** |  | **Durbin Conover** |  |
| --- | --- | --- | --- | --- | --- |
|  | | ***χ²* (*df*)** | ***P*** | ***t*** | ***P*** |
|  | |  |  |  |  |
| **Consumption occasions** | |  |  |  |  |
|  | In-between meals | 116.00 (1) | <.001 | 14.40 | <.001 |
|  | Replacement meal | 97.50 (1) | <.001 | 12.40 | <.001 |
|  | Other | 7.51 (1) | .006 | 2.78 | .006 |
|  | |  |  |  |  |
| **Physical context** | |  |  |  |  |
|  | |  |  |  |  |
|  | At Home | 140.00 (1) | <.001 | 17.20 | <.001 |
|  | Out of Home | 46.40 (1) | <.001 | 7.48 | <.001 |
|  | |  |  |  |  |
| **Social Context** | |  |  |  |  |
|  | |  |  |  |  |
|  | Alone | 122.00 (1) | <.001 | 15.00 | <.001 |
|  | With Others | 68.60 (1) | <.001 | 9.63 | <.001 |
|  | |  |  |  |  |
| **Temporal context** | |  |  |  |  |
|  | |  |  |  |  |
| **Dayparts** | |  |  |  |  |
|  | |  |  |  |  |
|  | Morning | 7.67 (1) | .006 | 2.81 | .005 |
|  | Afternoon | 161.00 (1) | <.001 | 20.30 | <.001 |
|  | Evening/Night | 53.00 (1) | <.001 | 8.13 | <.001 |
|  | |  |  |  |  |
| **Day types** | |  |  |  |  |
|  | |  |  |  |  |
|  | Weekdays | 22.70 (1) | <.001 | 4.98 | <.001 |
|  | Weekend Days | 84.20 (1) | <.001 | 11.10 | <.001 |
|  | |  |  |  |  |
| **Season** | |  |  |  |  |
|  | |  |  |  |  |
|  | Spring | 58.10 (1) | <.001 | 8.61 | <.001 |
|  | Summer | 108.00 (1) | <.001 | 13.40 | <.001 |
|  | Autumn | 56.60 (1) | <.001 | 9.33 | <.001 |
|  | Winter | 73.00 (1) | <.001 | 10.00 | <.001 |

**Table S11.** Perceived snacking occasions Daily Average Frequencies (DAFs) pairwise comparisons.

| **Comparisons** | **Friedman** |  | **Durbin Conover** |  |
| --- | --- | --- | --- | --- |
|  | ***χ²* (*df*)** | ***P*** | ***t*** | ***P*** |
|  |  |  |  |  |
| **Beverages as snack** | 417.00 (2) | <.001 |  |  |
| In-bet. vs. Repla. |  |  | 39.47 | <.001 |
| In-bet. vs. Other |  |  | 37.32 | <.001 |
| Repla. vs. Other |  |  | 2.15 | .032 |
|  |  |  |  |  |
| **Foods as snack** | 435.00 (2) | <.001 |  |  |
| In-bet. vs. Repla. |  |  | 37.38 | <.001 |
| In-bet. vs. Other |  |  | 46.89 | <.001 |
| Repla. vs. Other |  |  | 9.51 | <.001 |
|  |  |  |  |  |
| **All snacks** | 414.00 (2) | <.001 |  |  |
| In-bet. vs. Repla. |  |  | 36.58 | <.001 |
| In-bet. vs. Other |  |  | 38.86 | <.001 |
| Repla. vs. Other |  |  | 2.29 | .023 |
|  |  |  |  |  |
